# Implementation and Evaluation of COVIDCare@Home, a Family Medicine–Led Remote Monitoring Program for Patients With COVID-19: Multimethod Cross-sectional Study

**DOI:** 10.2196/35091

**Published:** 2022-06-28

**Authors:** Celia Laur, Payal Agarwal, Kelly Thai, Vanessa Kishimoto, Shawna Kelly, Kyle Liang, R Sacha Bhatia, Onil Bhattacharyya, Danielle Martin, Geetha Mukerji

**Affiliations:** 1 Institute for Health System Solutions and Virtual Care (WIHV) Women's College Hospital Toronto, ON Canada; 2 Department of Family and Community Medicine University of Toronto Toronto, ON Canada; 3 Women’s College Hospital Toronto, ON Canada; 4 Population Health and Values Based Health Systems Ontario Health Toronto, ON Canada; 5 Temerty Faculty of Medicine University of Toronto Toronto, ON Canada; 6 Peter Munk Cardiac Centre University Health Network Toronto, ON Canada; 7 Institute of Health Policy, Management and Evaluation Dalla Lana School of Public Health University of Toronto Toronto, ON Canada

**Keywords:** virtual care, COVID-19 pandemic, remote monitoring programs, social determinants of health, digital health, COVID-19, pandemic, health care, remote monitoring, clinical outcome, patient, health care cost, patient experience

## Abstract

**Background:**

COVIDCare@Home (CC@H) is a multifaceted, interprofessional team-based remote monitoring program led by family medicine for patients diagnosed with COVID-19, based at Women’s College Hospital (WCH), an ambulatory academic center in Toronto, Canada. CC@H offers virtual visits (phone and video) to address the clinical needs and broader social determinants of the health of patients during the acute phase of COVID-19 infection, including finding a primary care provider (PCP) and support for food insecurity.

**Objective:**

The objective of this evaluation is to understand the implementation and quality outcomes of CC@H within the Quadruple Aim framework of patient experience, provider experience, cost, and population health.

**Methods:**

This multimethod cross-sectional evaluation follows the Quadruple Aim framework to focus on implementation and service quality outcomes, including feasibility, adoption, safety, effectiveness, equity, and patient centeredness. These measures were explored using clinical and service utilization data, patient experience data (an online survey and a postdischarge questionnaire), provider experience data (surveys, interviews, and focus groups), and stakeholder interviews. Descriptive analysis was conducted for surveys and utilization data. Deductive analysis was conducted for interviews and focus groups, mapping to implementation and quality domains. The Ontario Marginalization Index (ON-Marg) measured the proportion of underserved patients accessing CC@H.

**Results:**

In total, 3412 visits were conducted in the first 8 months of the program (April 8-December 8, 2020) for 616 discrete patients, including 2114 (62.0%) visits with family physician staff/residents and 149 (4.4%) visits with social workers/mental health professionals. There was a median of 5 (IQR 4) visits per patient, with a median follow-up of 7 days (IQR 27). The net promoter score was 77. In addition, 144 (23.3%) of the patients were in the most marginalized populations based on the residential postal code (as per ON-Marg). Interviews with providers and stakeholders indicated that the program continued to adapt to meet the needs of patients and the health care system.

**Conclusions:**

Future remote monitoring should integrate support for addressing the social determinants of health and ensure patient-centered care through comprehensive care teams.

## Introduction

Remote home monitoring has dramatically expanded to manage COVID-19 in the community and avoid unnecessary hospital visits in a capacity-constrained health care system [1,2]. The ability to remotely monitor patients enables providers to escalate care at signs of deterioration, while minimizing the risk of unnecessary direct exposure of the general public, patients, and health workers to the virus [2,3]. Outcomes for remote home monitoring programs for COVID-19 are inconsistent but suggest low rates of mortality, admission rates, emergency department (ED) attendance, or reattendance [2]. Further, models of care delivery for remote monitoring vary significantly. Although many are implemented in specialist care settings [4-24], family medicine–led models may provide advantages, such as being more adaptable to meet evolving patient needs, including addressing psychosocial needs and social determinants of health within a limited capacity system [2].

To understand the impact of remote monitoring programs, evaluations of process and outcome measures are needed [25]. Greenhalgh et al [25] suggest that evaluations of COVID-19 remote monitoring programs should focus not only on the efficacy of monitoring respiratory symptoms but also on the evaluation of cost-effectiveness, patient experience, equity, sustainability, and adaptation [25]. To date, few evaluations have taken this comprehensive approach [2,16]. The Quadruple Aim framework of patient experience, provider experience, cost, and population health focuses on key process and outcome measures and is suggested as a set of principles for health system reform to be used worldwide [26].

The aim of this study was to conduct a comprehensive evaluation of the first 8 months of COVIDCare@Home (CC@H), a remote monitoring program, based at Women’s College Hospital (WCH) in Toronto, Canada, that aims to address the clinical and socioeconomic needs of patients during the acute phase of COVID-19. A detailed description of the program is provided separately [27]. Lessons from this program may be more broadly applicable to the use of remote monitoring for other acute and chronic conditions and are thus highly amenable to a primary care/family medicine approach [2]. The objectives of this evaluation are to understand the implementation and quality outcomes of CC@H within the Quadruple Aim framework of patient experience, provider experience, cost, and population health [26].

## Methods

### Study Design

This multimethod cross-sectional evaluation followed the Quadruple Aim framework [26] of patient experience, provider experience, cost, and population health, focusing on key process and outcome measures. Process measures included implementation outcomes of feasibility and adoption. Within patient and provider experience, the quality measures of safety, effectiveness, equity, and patient centeredness were assessed based on the National Academy of Medicine’s (formerly the Institute of Medicine) domains of quality [28]. Measures were selected based on applicability to the program and feasibility of data collection. The outcome of population health included stakeholder interviews to reflect on program and health system sustainability.

### Setting and Context

**Figure 1:**
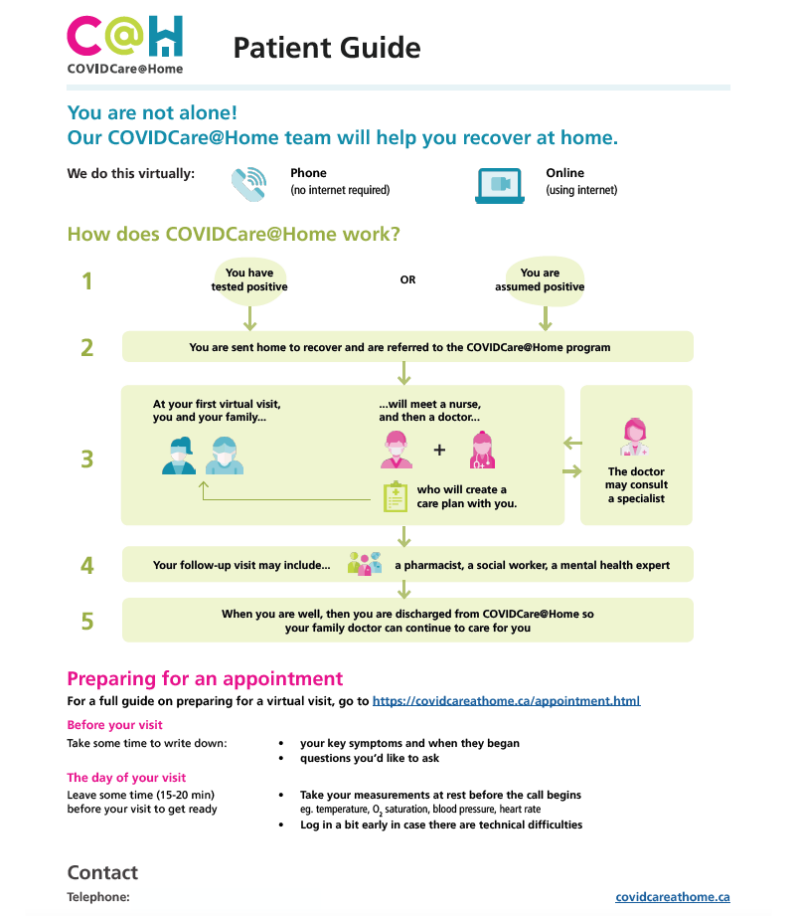
Patient guide to the CC@H program. CC@H: COVIDCare@Home.

CC@H was launched on April 8, 2020, and is based at the WCH, an ambulatory hospital in Toronto, Ontario, Canada. Adaptive leadership and ongoing improvement cycles were used to adapt the program to meet system needs as the pandemic evolved [29]. An in-depth description of the strategies used to adapt the program and additional contextual factors are provided elsewhere [29], as are details about the model of care [27]. A patient-facing outline of the program is provided in Figure 1. In brief, the program was led by an interdisciplinary primary care team, with support from multiple specialists and allied health members. Patient were monitored at home by phone or video, sometimes with the use of a pulse oximeter.

### Participant Recruitment

The program aims to support home-based patients with COVID-19. This study included all patients who had their first appointment in the first 8 months of the program, from April 8 to December 8, 2020. The primary referral source was the COVID-19 assessment center at the WCH. Patients were also referred by the assessment centers, ED, and acute care wards of neighboring health systems. Referred patients were excluded if they did not have access to a phone. In October 2020, to accommodate rising case numbers, individuals aged 20-40 years who had a primary care provider (PCP) were excluded.

### Outcomes

**Figure 2:**
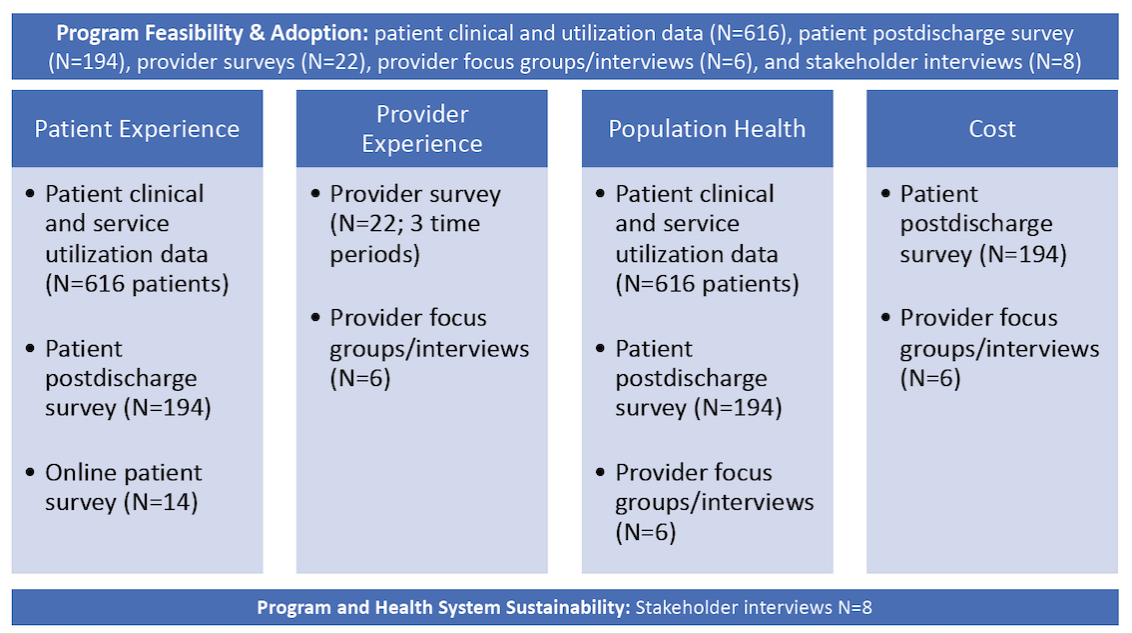
Summary of data sources and sample sizes within the Quadruple Aim framework for the CC@H evaluation. CC@H: COVIDCare@Home.

Results were organized by the Quadruple Aim framework of patient experience, provider experience, cost, and population health [26]. Process outcomes included feasibility and adoption. Within patient and provider experience, key quality measures included safety, effectiveness, equity, and patient centeredness. Data sources, samples sizes, and outcomes are summarized in Figure 2.

### Data Sources

A pragmatic approach was taken for data collection, with the aim to learn from all sources of data available. For this reason, various sources were used with variable sample sizes (Multimedia Appendix 1).

#### Patient Clinical and Utilization Data

Clinical information about participants was extracted from the electronic medical record (EMR) system (Epic, Epic Systems Corporation, Canada), including data entered in a standardized flowsheet (Multimedia Appendix 2). EMR data included age, sex, visit type and frequency, and length of time in the program. Flowsheet data included clinical characteristics, COVID-19–related characteristics, COVID-19 risk factors, and access to a PCP.

#### Patient Postdischarge Survey

Approximately 2 weeks after a patient was discharged, they received a standardized follow-up call from a nurse practitioner (NP), who asked questions verbally and entered data, including reflections and updates, into a standardized electronic flow sheet (Multimedia Appendix 3).

#### Online Patient Survey

An online patient evaluation survey (Multimedia Appendix 4) was developed with input from 2 lived-experience advisors. The survey was initially drafted by 2 researchers with experience in digital health evaluation, and then sent to 2 lived-experience advisors for written feedback. Once this feedback was addressed, a call was conducted between the researchers and the 2 advisors to work through each question and confirm wording and questions to add/remove, ensuring the importance and clarity of each question. Multiple scales were used in the survey as these were selected based on the information that was most useful to the program, rather than consistency of results.

Patients who consented to be contacted at the end of the postdischarge appointment were contacted by email to complete the survey administered through Research Electronic Data Capture (REDCap), a secure, web-based software platform [30], and for their responses to be linked to the extracted clinical and utilization data using their medical record number (MRN). Surveys were sent in 2 rounds, with the first open from July 30 to August 30, 2020, and the second from November 23 to December 8, 2020. Two reminder emails were sent in each round. All participants got an opportunity to complete the survey over the phone, and a translator was available for those who preferred to answer in a language that was not English. Due to a low response rate, after the second round, patients who had provided a valid phone number received a call from a research assistant in January 2021 to complete the survey by phone.

#### Provider Surveys

Brief provider surveys were developed by researchers in the study team and piloted with 2 individuals who were part of the study and providers in the program. The final version was administered through Qualtrics (Multimedia Appendix 5) [31]. This voluntary survey was emailed to all CC@H providers at 3 time points (round 1: June 24, 2020; round 2: August 24, 2020; round 3: December 2, 2020), with an email reminder 1 week later. Providers included physicians, social workers/mental health professionals, nurses, NPs, and pharmacists.

#### Provider Interviews and Focus Groups

All providers were given the option to participate in a virtual interview or focus group. Interviews and focus groups were conducted in July 2020 by a research assistant following a semistructured guide (Multimedia Appendix 6) regarding their experience, perceived patient experience, and the impact on the health system. Each discussion was audio-recorded then transcribed verbatim by a third party.

#### Stakeholder Interviews

Stakeholders, including managers and senior leadership involved in CC@H development, were recruited to participate in a semistructured, one-to-one, virtual interview. Participants were recruited by email between August 17 and October 8, 2020, with interviews conducted by a postdoctoral researcher (author CL). Interview questions (Multimedia Appendix 7) focused on health system impact, also addressing program feasibility and adoption, safety, equity, effectiveness, and patient centeredness.

#### Population Health: Ontario Marginalization Index (ON-Marg)

Postal code data for all participants were extracted from Epic, and the Ontario Marginalization Index (ON-Marg) [32] was calculated. ON-Marg is a data tool used to illustrate levels of marginalization across the province and combines a wide range of equity indicators based on postal code and separated by quintile [32].

### Data Synthesis

For patient clinical and utilization data, descriptive analyses were conducted using R software (R Foundation for Statistical Computing), with continuous variables reported as medians (IQR) and categorical variables reported as percentages. Data were not normally distributed, so medians were used. Patient (online and postdischarge) and provider surveys were analyzed descriptively in Microsoft Excel, and the net promoter score was calculated [33]. For provider focus groups, provider interviews, and stakeholder interviews, each discussion was audio-recorded and then transcribed verbatim by a third party. Deductive content analysis was then conducted by 2 researchers (authors CL and VK) using NVivo 2020 (QSR International), mapping to implementation and service quality outcomes listed earlier within the Quadruple Aim framework. Double-coding was using to confirm results, and discrepancies were discussed with authors PA and GM. Merging of quantitative and qualitive results into the quality outcomes within the Quadruple Aim framework was an iterative process, with some data aligning with more than 1 outcome.

ON-Marg was used to provide a score to examine overall marginalization using a summated value ranging from 1 to 5, where 1 reflects low levels of marginalization and 5 reflects high levels of marginalization. The score was used to assess the percentage of underserved patients in the CC@H program, where underserved was considered as being from the most marginalized quintile (score of 5). Individual dimensions in the score include (1) *residential instability*, area-level concentrations of people who experience high rates of family or housing instability; (2) *ethnic concentration*, high area-level concentrations of people who are recent immigrants or people belonging to a “visible minority” group, as defined by Statistics Canada; (3) *material deprivation*, closely connected to poverty and referring to inability for individuals and communities to access and attain basic material needs; and (4) *dependency,* area-level concentrations of people who do not have income from employment [32]. The ON-Marg analysis includes appointments of all statuses (completed, cancelled, etc) and repeat cases. Standard practices for calculating this score were used [32].

### Ethical Considerations

This study was completed by the investigators without the influence of any commercial sponsor and was approved by the local research ethics board at the WCH (2020-0058-E).

## Results

### Demographics

Clinical and service utilization data were collected for all patients in the first 8 months of the program (N=616). Of the 616 patients, 337 (55%) were female, the median age was 35 (IQR 25) years, and 171 (28%) did not have a PCP. The patient postdischarge survey was conducted 2 weeks postdischarge (N=194). Of these 194 patients, 110 (57%) were female and the median age was 35 (IQR 25) years. The online patient survey was completed by N=14 patients, who had a median age of 33 (IQR 21) years and 9 (64%) of whom were female (Table 1).

Providers who completed the survey (N=22, over 3 time periods) were majorly female and represented a diverse set of clinical roles. We conducted 3 interviews and 1 focus group were conducted with CC@H clinicians (n=6, 27.3%, no physicians). Stakeholders (n=8, 36.4%; 4, 50%, female) who participated in the interviews were in managerial or leadership roles at the WCH. Multimedia Appendix 8 includes the full tables of demographics and results for patients, separated by data collection tool. Multimedia Appendix 9 includes the demographics and results for providers.

**Table 1:** Demographic and clinical characteristics of all patients (N=616) admitted to the CC@H^a^ program from April 8 to December 8, 2021.

Clinical and service utilization data		Patients/visits
**Age (years), median 35 (IQR 25) years, n (%)**		
	Under 18 years of age	23 (3.7)
	Over 60 years of age	85 (13.8)
	Missing	508 (82.5)
**Sex, n (%)**		
	Male	279 (45.3)
	Female	337 (54.7)
**Comorbidity, n (%)**		
	Asthma	41 (6.7)
	Diabetes	36 (5.8)
	Hypertension	34 (5.5)
	Anxiety/depression	33 (5.4)
	Other (diabetes, hypertension, etc)	45 (7.3)
	Missing	427 (69.3)
**Has a PCP^b,c^, n (%)**		
	Yes	357 (58.0)
	No	171 (27.8)
	Missing	88 (14.2)
**Visits (N=3412), n (%)**		
	Generic provider	689 (20.2)
	Family physician staff/resident	2114 (62.0)
	Registered nurse	439 (12.9)
	Advanced nurse	2 (0.1)
	Social worker/mental health professional	149 (4.4)
	Pharmacist	19 (0.6)
Visits per patient, median (IQR)		5 (4)
Time from swab results to first visit, median (IQR)		3 (3)
Length of follow-up in program^d^, median (IQR)		7 (27)

^a^CC@H: COVIDCare@Home.

^b^PCP: primary care provider.

^c^Can select more than one option.

^d^Time from the first appointment to the last.

### Process Outcomes: Feasibility and Adoption

Based on the utilization data (Table 1), a total of 3412 visits were conducted in the first 8 months for 616 patients, including 2114 (62.0%) visits with family physician staff/residents and 149 (4.4%) visits with a social worker/mental health professional. There was a median of 5 (IQR 4) visits per patient, with a median length of follow-up of 7 days (IQR 27). The median time from a positive swab result to the first visit was 3 days (IQR 3). All visits were conducted by phone or video, with no in-person visits.

Within the patient experience data from the patient postdischarge survey (see Multimedia Appendix 8 for full results), 177 (91.2%) of 194 patients were referred from the WCH assessment center. During the program, 39 (20.1%) patients reported they received a pulse oximeter and 14 (7.2%) received a thermometer. In addition, 60 (30.9%) patients reported receiving a referral to a social worker. Within the patient survey data (Table 2), 11 (79%) of 14 patients reported that scheduling their remote visit was easy.

From the provider survey (see Multimedia Appendix 9), most providers at each time point did not have prior experience with remote monitoring programs. All but 2 (9%) of the 22 providers (all rounds) strongly agreed/agreed that they felt more comfortable with remote monitoring now than they did when they started the program. All but 5 (23%) providers (all rounds) strongly agreed/agreed they felt more comfortable with the technology involved in remote monitoring than when they started.

Provider interviews indicated that the initial development of the program was primarily physician driven and that involvement of nursing and allied health providers in decision-making grew as the program developed. Providers commented on the steep learning curve of rapid onboarding to a new program, adapting to delivering virtual care, using a new EMR system, challenges defining their roles and responsibilities, and getting used to the rapid decision-making needed to develop and adapt the program to meet changing patient and health system needs. Even given these challenges, all providers recognized a strong need for the program and understood there would be challenges when developing a program so rapidly during a pandemic.

Additional facilitators mentioned in stakeholder interviews included senior leadership support, resourcing, and having regular communication between experienced clinical, operational, and technological leads. The continuous research and evaluation approach also allowed for iterations of the program, which ultimately improved care delivery. For long-term effectiveness, stakeholders valued the interdisciplinary collaboration between physicians of a variety of disciplines (ie, primary care, internal medicine), allied health professionals, academic leaders, and information management/information technology (IM/IT).

**Table 2:** Online patient survey data (N=14): Detailed information collected through the online patient survey focused on feasibility, adoption, safety, effectiveness, patient centeredness, and health system connection and impact. A sample of questions have been selected here, with the full results provided in Multimedia Appendix 8.

Survey questions			Strongly agree, n (%)		Agree, n (%)		Neutral, n (%)		Disagree, n (%)		Strongly disagree, n (%)		N/A^a^, n (%)
**Safety**													
	I feel my COVID-19 infection was well treated.	7 (50)		3 (21)		4 (29)		0		0		0	
	The health care providers had a good understanding of my medical problem(s).	7 (50)		4 (29)		2 (14)		1 (7)		0		0	
	I feel my care was increased when needed.	5 (36)		4 (29)		1 (7)		1 (0)		0		3 (21)	
	The program helped me decide if/when I needed in-person medical care.	5 (36)		4 (29)		0		0		1 (7)		4 (29)	
	The program helped me avoid going to the ED^b^. (*Note: no patient who answered the survey went to the hospital.*)	8 (57)		3 (21)		3 (21)		0		0		0	
**Effectiveness**													
	The program helped me to better manage my health and medical needs for COVID-19.	8 (57)		3 (21)		2 (14)		1 (7)		0		0	
	I feel I had enough time with the doctor(s).	7 (50)		5 (36)		2 (14)		0		0		0	
	I feel I had enough time with the other providers (ie, nurse, social worker, etc).	4 (29)		5 (36)		4 (29)		1 (7)		0		0	
**Patient centeredness**													
	I feel the care I received is in line with my goals and preferences.	7 (50)		6 (43)		0		1 (7)		0		0	
	This program eased my anxiety immediately after my positive COVID test.	7 (50)		4 (29)		2 (14)		1 (7)		0		0	

^a^N/A: not applicable.

^b^ED: emergency department.

### Patient Experience

#### Equity

Of the 839 patients available in the ON-Marg data, 195 (23.2%, range 95-317, 11.3%-37.8%) were completed by patients in the most marginalized populations (marginalization score=5). Within the most marginalized, the median was 79.7% for residential instability, 74.4% for ethnic concentration, 40.4% for deprivation, and 14% for dependency.

When analyzed by visit, 564 (24.4%) of 2316 visits (range 257-831, 11.1%-35.9%) were completed by patients in the most marginalized populations. Within those most marginalized, the median by visit was 77.2% for residential instability, 75.9% for ethnic concentration, 37.6% for deprivation, and 15.8% for dependency.

#### Effectiveness

In the online patient survey, the net promoter score was 77 [33]. Of the 14 patients, 11 (79%) strongly agreed/agreed that the program helped them to better manage their health and medical needs for COVID-19 and agreed that the program was useful for managing their care and treatment (Multimedia Appendix 8). In the patient postdischarge survey data, when asked about the most helpful part of the program, 69 (35.6%) of 194 patients appreciated the regular check-ins and 48 (24.7%) mentioned a positive care experience. Many patients mentioned they felt supported and reassured and that they received comprehensive, timely, and personalized care during a challenging time. Details are provided in Multimedia Appendix 8.

#### Safety

Of the 194 patients, 10 (5.2%) in the patient postdischarge survey (see Multimedia Appendix 8) reported that since they had been diagnosed with COVID-19, they had accessed emergency services, including the ED, for COVID-19 or any other health issues. In addition, 117 (60.3%) patients felt they were discharged from CC@H at the right time and only 6 (3%) and 2 (1%) felt they were discharged too early or too late, respectively (n=70, 36%, were missing data).

Within the online patient survey data (Multimedia Appendix 8), 10 (71%) patients strongly agreed/agreed that their COVID-19 infection was well treated; 9 (64%) strongly agreed/agreed that their care was increased, when needed; and 9 (64%) strongly agreed/agreed that the program helped them decide if/when they needed in-person medical care.

#### Patient Centeredness

In the online patient survey data (Multimedia Appendix 8), 11 (79%) of 14 patients strongly agreed/agreed that the program eased their anxiety immediately after their positive COVID-19 test and throughout the program and 13 (93%) patients agreed/strongly agreed that their needs were addressed in the program. All but 1 (7%) patient agreed that the care they received was in line with their goals and preferences.

### Provider Experience

#### Equity

Provider survey results (see Multimedia Appendix 9) found that all but 1 (10%) of 10 provider (round 1; 4, 40%, neutral) agreed/strongly agreed that they were able to address issues around social determinants of health for their patients. In addition, 14 (64%) of 22 providers (all 3 rounds) agreed/strongly agreed that the program was meeting the needs of underserved populations. Provider interviews demonstrated mixed opinions on whether the program initially met the needs of underserved populations, mainly focusing on the steep learning curve of navigating patients with varying immigration statuses (eg, refugees, undocumented immigrants) due to a lack of experience. Providers reported challenges in finding community resources that still offered social services throughout the COVID-19 pandemic. Resources and expertise from Crossroads Clinic, a WCH clinic specializing in refugee care, helped providers better support undocumented patients and thus improved the quality of the program [34].

#### Effectiveness

Interviewed providers generally agreed that CC@H was effective and met the needs of its patients. Provider interviews also indicated provider and program flexibility were invaluable when responding to the changing environment. The primary care model and flexibility of the staffing and resources meant the team could provide comprehensive care outside the COVID-19 diagnosis to holistically support the needs of their patients. For example, due to this flexibility, a patient was able to continue within the program despite no longer presenting COVID-19 symptoms, because they required medical care but did not have access to a PCP.

We had one homeless gentleman who also had prostate cancer. He wasn’t diagnosed with us, but he didn’t have a family doctor. We followed him until we were able to have him see a family doctor because we got him a family doctor, but they couldn’t see him for another month and a half, so we just kept monitoring him. So, there’s a flexibility there to accommodate the needs of everyone.

Health care provider 1

#### Safety

All but 1 (5%) provider (see Multimedia Appendix 8; 3, 14%, neutral) agreed/strongly agreed that they felt supported to manage the clinical uncertainty of a new illness. All but 1 (5%; 1, 5%, neutral) provider agreed/strongly agreed they could escalate patient care, when needed. All providers (4, 18%, neutral) felt the program helped to avoid ED visits. Provider interviews discussed how frequent communication within the team about evidence and program changes was initially conducted through daily interdisciplinary group huddles to discuss patient safety and clinical issues. The primary care approach was also said to make CC@H better equipped to adapt to clinical uncertainty compared to other specialties, and thus increased the safety of the program.

In order to work in that setting [primary care], you have to be comfortable with a level of uncertainty…it’s just their ability to kind of embrace the uncertainty of “you may not know the diagnosis and that’s OK in family medicine.” I think that’s why this group of physicians was really ideally poised to take this on, because they do that every day.

Health care provider 2

#### Patient Centeredness

Although patient needs varied within the program, interviewed providers generally felt that CC@H was able to improve access to medical, mental health, and social care. Specifically, they agreed that the program helped to ease patient anxiety regarding a positive COVID-19 diagnosis. Providers also commented that patients valued receiving care specific to and beyond their positive COVID-19 diagnosis, which helped to relieve their anxiety. All providers agreed/strongly agreed (3, 14%, neutral) that they could provide patient-centered care through the program, and all but 2 (9%; 3, 14%, neutral) agreed/strongly agreed that the care they could provide through the program aligned with the goals and preferences of their patients.

#### Population Health

Population health was explored as patient demographics (comorbidities and smoking status), access to a PCP, and receipt of community support. Utilization data indicated the most common comorbidity was asthma (n=41, 6.7%), followed by diabetes (n=36, 5.8%), hypertension (n=34, 5.5%), and anxiety/depression (n=33, 5.4%). In addition, 47 (7.6%) patients were smokers.

In the patient postdischarge survey (Multimedia Appendix 8), 32 (16.5%) of 194 patients were connected to a PCP by the program if they did not have one when entering the program. Other community support provided by CC@H included the following: 9 (4.6%) patients received food delivery, 4 (2.1%) were connected to the Red Cross, and 4 (2.1%) were connected to other types of support, such as government financial support, counseling resources, laundry, and pharmacy delivery.

In the patient postdischarge survey (Multimedia Appendix 8), when asked where they would have gone after their diagnosis if they were not involved in CC@H, 33 (17%) patients said they would go to a PCP, 17 (8.8%) said they would not have sought care, and 14 (7.2%) would have gone to the ED. From the online patient survey data (Multimedia Appendix 8), 8 (57%) patients would have gone to their PCP and 4 (29%) to the ED. When asked how many in-person visits they thought they would have had to make to a health care provider, the mean was 3 (SD 6.7, range 0-25) visits. In addition, 10 (71%) patients strongly agreed/agreed that the program could be beneficial for other patients with a lot of health issues.

Provider interviews indicated that 1 of the most valuable components of CC@H was finding PCPs for patients who did not have one. Beyond medical care, providers also reported the program was able to support patients to access groceries and medication and assist them with accessing government financial support.

#### Program and Health System Sustainability

CC@H stakeholder interviews focused on the sustainability of CC@H and future plans for remote monitoring. Facilitators for the sustainability of CC@H during the COVID-19 pandemic included the family medicine interdisciplinary model and having the flexibility to scale resources up and down, as needed. These facilitators were also said to support the sustainability of the health system by providing comprehensive care to patients beyond their COVID-19 diagnosis, while minimizing the risk of direct exposure to the public, patients, and health workers. Stakeholders indicated that comprehensive physician remuneration and billing codes are needed to incentivize the remote monitoring care model to improve the sustainability of remote monitoring programs in general.

Beyond the COVID-19 pandemic, stakeholders mentioned that the program could be adapted to other areas and be used to create a set of remote monitoring principles.

We have an opportunity as an organization to understand and learn from the experience that the program has with these [remote monitoring] tools…I also was always conscious of the potential for adaptation of a program like this into very [low-resource] environments whether that be in the far north or whether it be outside our borders.

Stakeholder 8

Stakeholders also commented on the Quadruple Aim framework, emphasizing the importance of equitable care in the current and future iterations of the program. Comments aligned with previously mentioned experiences, with an additional point on the benefit of having diverse staff supporting diverse patients.

The [medical] residents that were engaged in the team were also very diverse, so their shared experience was helpful. In one situation we had a Black woman who really related to the fears and concerns of another Black woman who happened to have COVID, so by having a diverse group of caregivers that has also, I think, enriched the program.

Stakeholder 4

#### Cost

Within the Quadruple Aim framework, low ED visits with patients can be considered proxy for cost avoidance [35]. As mentioned above, 10 (5%) patients from the post-discharge survey reported that since they had been diagnosed with COVID-19, they had accessed emergency services, including the ED, for COVID-19 or any other health issues. From the provider survey, all providers agreed/strongly agreed (4, 18%, neutral) the program has helped avoid ED visits. Interviewed providers and stakeholders perceived that CC@H prevented ED and intensive care unit (ICU) admissions.

Online patient survey data found that 3 (21%) of the 14 patients reported that they would have spent more than CA $300 (US $ 231.35) per visit on traveling to a health care provider (eg, parking, transit), missing work and other expenses (eg, childcare); see Multimedia Appendix 8.

Provider interviews indicated that remote monitoring services have the potential to save health system costs by decreasing ED visits and hospitalizations. Stakeholder interviews discussed how crucial the implementation of the virtual billing codes is to enabling physicians to be involved in the program.

## Discussion

### Principal Findings

Evaluation of the CC@H remote monitoring program using the Quadruple Aim framework found the program can provide safe, effective, and patient-centered care for patients with COVID-19. With 3412 visits conducted in the first 8 months and a net promoter score of 77, the program was feasible, with care provided to a wide demographic range of patients, using primarily phone, video, and remote monitoring devices, including pulse oximeters. Our results indicate multiple benefits of remote monitoring, particularly related to patient experience. Patients highlighted the value of a continuous, hands-on touchpoint; reassurance through regular check-ins; and support in addressing the social determinants of health, including access to food, medication delivery, and a PCP.

There is also evidence that the program design enabled more equitable care, allowing the program services to reach those who were disproportionately impact by COVID-19. In this study, 23.3% of the patients were in the most marginalized quintile patient population, suggesting an overrepresentation of patients from low-socioeconomic-status groups. This 23% is higher than the median 17.6% across the WCH.

In CC@H, 28% of the patients did not have a PCP, which is much higher than the 2019 level in Ontario, which was 9.4% (14.5% across Canada) [36]. This equity focus is especially important for COVID-19 care, as we know patients from these communities were disproportionately impacted by the disease [37-39]. The positive impact on patient-centered care and equity may reflect the comprehensive, family medicine–led, team-based design of the program.

Despite the rapid development and limited experience by providers in remote monitoring or treating patients with COVID-19, most interviews with providers and stakeholders indicated that support from leadership and team flexibility made them feel comfortable and allowed for continuous adaptions to meet evolving patient and health system needs. Almost all providers and patients felt the program helped to avoid unnecessary ED visits.

### Comparison With Prior Work

Over the course of the pandemic, there has been a rapid growth in the use of remote monitoring programs to support patients and health systems. However, there is significant variation in program design and patients served (range 12-6853 patients per program), and evaluations of these programs and their impact are limited [2]. Most other programs described in the literature did not take a comprehensive, family medicine–led, team-based approach. Programs were either specialist led or focus on the use of technology as the primary mechanism for daily check-ins [4-24].

A review of COVID-19 remote monitoring programs found that few program models included support of mental health [2]. Evaluations of most programs focused on reporting adoption data (ie, number of visits) and basic clinical outcomes (eg, ED visits, deaths), with limited data presented on impact across the Quadruple Aim framework or on equity [2]. This CC@H evaluation is comprehensive, including reporting on patient, provider, and stakeholder perspectives, and assesses impact on delivery of equitable care [2]. When comparing implementation outcomes, other programs had a virtual length of stay ranging from 3.5 to 13.1 days [2] compared to CC@H’s median of 7 days. Time from swab to assessment ranged from 2 to 3.7 days [2], similar to CC@H’s median of 3 days. Mortality rates were also similar, ranging from 0% to 3.1% in other programs, with admission or readmission rates ranging from 0% to 29%. ED attendance or reattendance ranged from 4% to 36%, while in CC@H, it was 5%.

Most previous studies of remote monitoring programs, primarily related to cardiac disease, failed to evaluate impact of the program on patient experience or quality of life [40-42]. Further, almost none looked specifically at the impact on social determinants of health or patients from underserved populations [40-42]. Our results suggest that future remote monitoring programs beyond the pandemic might benefit from a comprehensive team-based approach that prioritizes patient experience and support for the social determinants of health, in addition to more traditional clinical outcomes.

### Limitations

This study used a pragmatic approach by leveraging the regular collection of quality improvement data, which enabled evaluation under the constraints of the pandemic; however, it also led to some issues with data quality, particularly for missing values. In data collected through EMRs, an empty response may have represented “no,” “not applicable,” or missing data, with no way to distinguish between these options. ED and hospital utilization data were only collected through the patient postdischarge survey based on patient reporting and may not reflect all ED visits. As providers did not always document additional services, such as support with food delivery, these values are likely lower than the actual care provided. We also were unable to collect information regarding the number of people who were excluded for not having access to a phone, as this would be an indicator of socioeconomic deprivation. Other programs at the WCH were set up to support these individuals without access to a phone. Due to our sample size, we were unable to stratify our results by waves of the pandemic.

The patient postdischarge survey data were collected by an NP as part of clinical care as it provided significant insight into the patient experience. However, data were not collected for all patients, nor were comments transcribed verbatim. This clinical approach meant data were not anonymous and were collected by someone involved in care delivery. Triangulation across multiple sources of patient experience data helped to limit the impact of all potential bias. Although this evaluation included many measures with small numbers, it is encouraging that all were pointing in the same direction, thus suggesting patient benefit. A full cost-effectiveness analysis was outside the scope of this study and deserves further exploration.

### Conclusion

The CC@H remote monitoring program at the WCH is feasible and provided equitable, effective, safe, and patient-centered care during the COVID-19 pandemic. The primary care approach is thought to have facilitated comprehensive care, supporting patient needs beyond the COVID-19 diagnosis. Future remote monitoring programs should emphasize patient experience and the role of flexible, comprehensive, interdisciplinary programs that specifically address the social determinants of health. Using the Quadruple Aim framework facilitates understanding the impact of the program beyond clinical outcomes to support delivery of comprehensive, patient-centered care for all patients.

## Appendix

Multimedia Appendix 1 COVIDCare@Home protocol.

Multimedia Appendix 2 Electronic flowsheet for clinical and service utilization data.

Multimedia Appendix 3 Patient postdischarge survey.

Multimedia Appendix 4 Online patient survey.

Multimedia Appendix 5 Provider survey and digital consent.

Multimedia Appendix 6 Provider interview guide.

Multimedia Appendix 7 Stakeholder interview guide.

Multimedia Appendix 8 Full tables of all demographics, clinical characteristics, and results collected for patients.

Multimedia Appendix 9 Provider demographics and survey response focused on feasibility, adoption, safety, equity, effectiveness, patient centeredness, and cost.

## Abbreviations

CC@H: COVIDCare@Home
ED: emergency department
EMR: electronic medical record
NP: nurse practitioner
ON-Marg: Ontario Marginalization Index
PCP: primary care provider
WCH: Women’s College Hospital

## Data Availability

Data may be available upon request.
