# Supplementary material for: Implementation and Evaluation of COVIDCare@Home, a Family Medicine–Led Remote Monitoring Program for Patients With COVID-19: Multimethod Cross-sectional Study
Source: JMIR Hum Factors. 2022 Jun 28;9(2):e35091. doi: 10.2196/35091 (PMC9239565; doi:10.2196/35091)
Supplement: Multimedia Appendix 1 [file humanfactors_v9i2e35091_app1.pdf]

Study Protocol
Version Number: 4.0
Date: Sept 14, 2020

Developmental Evaluation of the COVIDCare@Home program for comprehensive team based remote monitoring

Evaluation Team

Co-Primary Investigators: Dr. Payal Agarwal & Dr. Geetha Mukerji

Primary Project Contact: Dr. Celia Laur
Research Assistant: Shivani Chandra
Program Manager: Ena Ujic
Research Assistant: Tanjeen Taha

Co-Investigators:

- Dr. Sacha Bhatia, (Scientist WCRI) - Executive sponsor
- Dr. Onil Bhattacharyya (WCH, WIHV, Senior Scientific Advisor)
- Dr. Nick Pimlott (Senior Advisor, WCH/Associate Chief ,WCH)
- Shawna Kelly (Nurse Practitioner, WCH)

Stakeholders/Steering Committee Members:
- Dr. Danielle Martin (EVP & Chief Medical Executive Officer)
- Elaine Goulbourne (Director, Primary Care, Clinical Resources and Performance)
- Dr. Ruth Heisey (Chief WCH Primary Care, Family Physician)
- Dr. Risa Freeman (Vice-chair, Education & Scholarship, DFCM University of Toronto)
- Dr Nikki Woods (University of Toronto)
- Dr. David Rojas (WCH)
- Darryl Yates, (VP Patient Care & Ambulatory Innovation) - Executive sponsor
- Dr. David Tannenbaum (Interim Chair, Department of Family and Community Medicine/DFCM)
- Dr. Pauline Pariser (Family Physician, SCOPE/UHN)
- Dr. Paula Harvey (Chief, Medicine, WCH)
- Dr. Simone Vigod (Chief, Psychiatry, WCH)
- Dr. Sheila Riazi (COVID Assessment Centre, WCH)
- Dr. Michelle Naimer (Associate Chief Family Med, MSH)
- Dr. Clarys Tirel (ED, Mount Sinai FHT)
- Dr. Luke Devine (MSH, GIM)
- Dr. Howard Ovens (MSH, ER
- Dr. Rebecca Stovel (WCH, GIM)
- Dr. Owen Lyons (WCH, Respirologist)
- Dr. Batya Grundland (DFCM, Associate Program Director, Curriculum & Remediation)

Background

The COVID-19 pandemic has created an unprecedented shift in the health care system. As demand on acute care resources including emergency departments and inpatient beds increases, most symptomatic yet stable patients will be required to monitor their COVID-19 symptoms at home.

The goal of the **COVIDCare@Home** project is to support primary care providers in caring for their patients with COVID-19 from their homes and supporting patients with COVID-19 with limited access to a primary care provider.

The program will:

- ***Enhance access***: Build practical pathways and tools (e.g. EMR based clinical flowsheets, digital self-management apps) to support primary care providers in virtually supporting their patients with COVID-19. This will enable evidence-based monitoring of patients and facilitate timely escalation if symptoms deteriorate. Links to acute care facilities will allow for rapid monitoring of post discharge patients recovering at home.
- ***Connect expertise:*** Support primary care in the form of a virtual COVID-19 support hub (housed at WCH) with specific resources to address medical, mental health and social needs that impact outcomes for COVID-19. This hub will provide real time support to PCPs and patients including expertise in GIM, nursing, social work, pharmacology and psychiatry.
- ***Provide care at a distance:*** Enable virtual care through phone or video with the potential for delivery of home monitoring devices such as a pulse oximeter to patients. This will include capacity to do remote monitoring from WCH should a PCP prefer for the DFCM team to provide this service, with meaningful handover and ongoing communication with the PCP.
- ***Support in person care***: Access to the WCH AACU as needed, to limit burden on acute care settings.

To evaluate this program, a developmental evaluation approach will be used to understand how the program to is able to rapidly learn, pivot and scale to meet the evolving needs of this pandemic.

There are multiple objectives to this developmental evaluation, allowing for consideration from a variety of perspectives. Objectives align with the quadruple aim to achieve the best possible health outcomes, improve the patient experience and workforce experience, while realizing the best use of resources.

Evaluation Objectives:

1. To use key process indicators (# of visits, # of referrals, etc.) to explore the rapid implementation and uptake of the COVIDCare@Home remote monitoring program, and how it changed over time.

2. To explore the barriers and enablers to the rapid development of a program that adapted to clinical, process and system uncertainty, including the roles of leaders, providers, and stakeholders in the decision-making and enactment processes.

3. To determine the short-term impact of COVIDCare@Home on:

    a) Patients including health outcomes and experience with COVID-19, health services utilization, and potential patient cost-savings of a remote monitoring program (i.e. savings from travel [parking, gas, public transit], childcare, work hours, and other costs)

    b) Providers' experience (including physicians, nurses, pharmacist and social work) delivering care to patient with COVID-19 using remote monitoring.

Methods

This developmental evaluation seeks to evaluate the implementation, service quality, and impact of the COVIDCare@Home program. The WIHV evaluation team is working alongside the COVIDCare@Home team, Women's Virtual operations team members, and clinic team members to enable collection of evaluation findings that will enable rapid iterative improvement cycles as the intervention is implemented. Findings from this evaluation will enable the intervention implementation to be adjusted rapidly as required, exploring the role of adaptive leadership within the implementation process.

Participants

COVIDCare@Home aims to support patients with COVID-19 (swab + or presumed) who are staying in their homes. Participants either have a Primary Care Provider (PCP) who is sharing the care with the COVIDCare@Home team, or have limited access to a PCP. All patients are eligible to participate in the patient experience interviews and a random selection of those who consent will be selected for a telephone interview. For randomization, every third patient who agrees to be interviewed will be contacted. If recruitment is low, it is possible that all patients who agree to an interview will be contacted. Providers of this service, including physicians, nurses (RNs), nurse practitioners (NPs), and other clinicians will all be invited to participate in surveys and/or focus groups and/or interviews.

Update: As participant recruitment is low, patient who agreed to participate and have not yet completed the survey, will receive a phone call from a Research Assistant to do a combined survey and interview over the phone.

Internal and External stakeholders will also be invited to participate in a one-to-one telephone interview. All internal stakeholders will be offered the opportunity of an interview, with reminders sent until saturation. After saturation, if an internal stakeholder still wishes to be interviewed, the interview will go ahead.

External stakeholders will be identified through the internal stakeholders and by internet searches of existing programs in Ontario. Examples of programs include the Connected COVID Care program at UHN, and the COVID-19 Expansion to Outpatients (COVIDEO) Program at Sunnybrook.

Sample Size Justification

All patients of the COVIDCare@Home program since its inception in April 2020 are eligible (n=200) and will be asked to participate in the evaluation. Those who consent to being contacted for evaluation of their experience will be sent a survey to capture their experience; 10-20 randomly selected patients (from within those who consent) will be selected to participate in an interview. Participants will be recruited until data saturation of themes from qualitative interviews is reached.

Update: As the patient survey response was low, and the number of patients in the program decreased, a combined survey/interview process will be followed.

All providers will have the opportunity to participate in the evaluation of their experience. There are approximately 20 internal stakeholders currently involved, however this number also fluctuates as the program develops.

Purpose sampling technique will be used for stakeholders who will be invited to participate in an interview. For internal stakeholders, interviews will be conducted with 5-10 individuals, who provide varying perspectives. For external stakeholders, 3-5 interviews will be conducted to get a basic understanding of the other programs.

Sample Size Summary

Patient Surveys: max n=200 Patients
Patient Interviews: n=10-20
Provider Surveys: ~ 20 x 4 surveys
Provider Interviews: 1 interview every two weeks until saturation
Physician Focus Groups: 2 total (5-7 people/FG)
NP/RN Focus Group: 1 total (5-7 people / FG)
Internal Stakeholders: n=5-10

External Stakeholders: n= 3-5

Data Collection

To meet the objectives, data will be collected from the following target populations through the described data collection methods:

1. ***Patient Surveys:*** During a clinical visit with the NP or RN near the end of their time with the program, all patients will be asked (Appendix 1) if they agree to receive more information about being involved in an evaluation to help improve the program. If agreed, NP/RN will document this in the chart, including if the participant preferred a telephone interview or needs an interpreter. Once per week, a data pull will be conducted (Appendix 18) of the recruitment information and provided to the Research Assistant. This data pull will only include those who answered yes to receiving more information. It will also include the patients name and Medical Record Number (MRN). The MRN is the only way to reliably link patient information to survey data using our method of data pulls from EPIC (details below).

The Research Assistant will send an e-mail (Appendix 2 and 3) with the consent for survey and chart review, as well as information about the interview, to participants who agreed. This email (Appendix 2) will include the survey link that has embedded digital consent. The e-mail will indicate that an RA can call them to talk through the process if further information is requested or to complete the survey over the phone. If an interpreter is needed, the survey will be conducted over the phone with the RA and the interpreter.

The e-mail will include a link to a RedCap survey, a secure data collection tool approved by the hospital, which will include digital consent before the survey questions are provided, including consent for linking the data. If phone is preferred, the RA will complete the verbal consent and survey over the phone, and will transfer the information into the RedCap survey, so all information remains in one place. If created, the written copy of responses will be destroyed as soon as it is entered into RedCap. Two reminder e-mails will be sent for those who requested e-mail follow-up, and 3 reminder phone calls for those who requested the survey by phone.

All questions will be asked in the survey in a way that the participant can skip any question.

Patient Interviews: At the end of the survey, the patient will be asked if they are interested in participating in a virtual interview to further explain their overall experience with the program. They will be informed that only randomly selected

patients will be contacted for an interview. If they click yes to the interview and are selected, the RA will contact the patients to arrange a time for the interview. Before the interview, a verbal consent process (Appendix 5a and b) will be used to ensure consent of the interview and remind them about the recorder and their option to decline any questions. Participants will have an opportunity to ask questions before beginning. The interview will focus on details of their experience in the program. The patient will be reminded not to provide identifying information about themselves or the providers. If they have a specific concern about an individual or the program, they will be e-mailed or mailed the pamphlet produced by Patient Relations with directions for submitting this specific information. Interviews will be stored on a secure drive on a WCH server, only accessible to key members of the evaluation team.

Patient Update: Due to a low response rate, after the second e-mail reminder to complete the survey is sent to patients who agreed to follow-up, each patient who agreed, yet has not completed the survey will receive a follow-up telephone call. This call will include a combined survey/interview to reduce participant burden. Calls will only be made after the final reminder is sent. Each patient will be called twice. After the second call, no further attempts will be made, unless requested by the participant. Consent will be taken at the beginning of each call, and the call recorded, if approved by the participant. The interview will still go ahead if the participant does not want to be recorded, and the RA conducting the call will take comprehensive notes.

2. ***Linking Patient Surveys to Chart Information:*** In the initial information e-mail and consent form (Appendix 2 & 3), participants will be provided information about the chart review that will link their survey responses to specific information in their chart. The specific information includes: age, sex, comorbidities, medications, if they are registered with a PCP (Yes/No), date of first / last appointment, total number of appointments, if an oximeter was sent, if they interacted with allied health (social worked, pharmacist etc.), and number of GIM consults. The letter will explain why this information is important for our evaluation. This linking process is necessary so as not to duplicated questions for participants to answer and also provides definitive information about number of appointments, how long they were in the program, and use of additional services.

   In the survey, before survey questions are asked, the digital consent will remind participants about this linking process. If participants do not consent to the survey, they will be thanked for their time and exited from the survey. As unlinked surveys do not provide enough information to understand the complete picture of the patient, only those who consent to data linkage will be asked to complete the survey.

   The information from the initial data pull will be securely kept on the WCH server in a secure drive, only accessible to key members of the research team. Upon receipt of that information, the MRN will be transferred to one master linking log that will be password

protected. The linking log will convert the MRN to a participant ID code. That code will be used by RedCap to send coded surveys, allowing the link back to EPIC details (Appendix 18).

Update: The MRN will still be used to link the call recordings to patient information, if agreed by the participant in the consent process.

3. Providers of COVIDCare@Home will be involved in the evaluation in several ways.

   a. ***Clinician surveys:*** A brief survey (Appendix 8) will be sent by secure e-mail (Appendix 6 &6) to all clinicians involved in the program, as soon as ethics is obtained, then 1, 3 and 6 months later (4 surveys total). This survey (Appendix 8) aims to monitor their changing perspectives over time as the program develops, including how well supported they feel and if they are meeting the needs of their patients. The survey will be administered through Qualtrics, a program supported by WCH. One reminder e-mail will be sent after one week. All questions will be asked in the survey in a way that the participant can skip any question.

   b. ***Physician Focus Group:*** Two virtual focus groups will be conducted with all available physicians involved in the program. Physicians will be e-mailed to request their participation in the focus group, and if they agree, will be asked to read the information letter and provide an email confirmation they have reviewed the consent and have agreed to participate (Appendix 17). Each focus group (Appendix 9) will take approximately 45 minutes and will focus on how the program responds to system, process and clinical uncertainty. Focus groups will be audio recorded then transcribed and files kept on a secure server at WCH only accessible to evaluation team members. Voluntary participation in the evaluation project will be emphasized within the consent document. The first focus group will occur shortly after ethics approval, and the second a few months later.

   c. **Physician Interview:** Once every two weeks for the first 2 months of the program, a physician will be interviewed about their experience with the program. Alongside the program evaluation, another team at WCH and the University of Toronto is exploring the educational experience of residents involved in this program. Their evaluation will be conducting separate data collection with residents, including interviews about their educational experience. To gain perspective on the educational impact of this program, instead of conducting a second interview, the educational team has added questions to the program evaluation interview guide. The recording and transcript from this interview will be used by both the program and the educational evaluation teams. This is explained as part of the consent form (Appendix 7). If physicians have any questions about the education project, they are to contact, Hollie Mullins, the Research Associate

on that project. This process has been confirmed with both teams in order to reduce the burden on physicians.

d. ***Nurse and Nurse Practitioner Focus Group:*** One virtual focus group will be conducted with all available RNs and NPs involved in the program. Participants will be e-mailed (Appendix to request their participation in the focus group, and if they agree, will be asked to read the information letter and sign the written consent form (Appendix 6) or send email confirmation they have read the consent and confirm voluntary participation. The focus group will take approximately 45 minutes and will focus on how the program responds to system, process and clinical uncertainty. Focus groups will be audio recorded then transcribed and files kept on a secure server at WCH. Voluntary participation in the evaluation project will be emphasized within the consent document.

e. ***Allied Health Professional Focus Group:*** One virtual focus group will be conducted with all available allied health professionals involved in the program. Participants will be e-mailed to request their participation in the focus group, and if they agree, will be asked to read the information letter and sign the written consent form (Appendix 6) or send email confirmation they have read the consent and confirm voluntary participation. The focus group will take approximately 45 minutes and will focus on how the program responds to system, process and clinical uncertainty. Focus groups will be audio recorded, transcribed and files kept on a secure server at WCH. Voluntary participation in the evaluation project will be emphasized within the consent document.

f. ***General Interviews:*** All providers involved in the program will have the option to participate in a one-to-one telephone interview, if they have not participated in a focus group or previous interview. Participants will be e-mailed to request their participation, and if they agree, will be asked to read the information letter and sign the written consent form (Appendix 6) or send email confirmation they have read the consent and confirm voluntary participation. If written consent is unavailable prior to the interviews, verbal consent will be taken. The interview will take approximately 30 minutes and will focus on how the program responds to system, process and clinical uncertainty. Interviews will be audio recorded and transcribed and files kept on a secure server at WCH. Voluntary participation in the evaluation project will be emphasized within the consent document.

4. ***Group Meeting Observation:*** Daily, 1-hour, virtual huddles/meetings are conducted with physicians, nurses, allied health and residents involved in the program. Once per week, a

member of the evaluation team will observe the huddles, and complete a standardized feedback recording sheet (Appendix 12) to take note of how decisions are made, and how the group adapts to system, process and clinical uncertainty. This observation process is described in the initial consent information for which clinicians will consent and at the beginning of each group meeting, the lead will inform the group that the observer is present and can be asked to leave at any time.

5. ***Internal Stakeholders:*** All stakeholders from WCH involved in the program will have the option to participate in a one-to-one telephone or Zoom interview.Women's College Hospital has recently launched the Office of Spread and Scale (OSS) that aims to support programs, such as COVIDCare@Home, to be spread to other settings or scaled more widely. As the OSS is providing direction to the COVIDCare@Home program, the interview will include questions about how you think the OSS can support this, and other programs. A consent form and information letter (Appendix 13) will be sent by e-mail by the Postdoctoral Researcher. If written consent is not provided, consent will be taken verbally ahead of the interview. Questions (Appendix 14) will focus on program development and how to cope with system, process and clinical uncertainty, as well as about how the OSS can support spread of C@H to other settings. All interviews will be audio recorded, transcribed and stored on a secure server at WCH, only accessible to the evaluation team.

6. ***External Stakeholders:*** Representatives from other organizations in Ontario who have set up similar remote monitoring programs for COVID-19 patients will be contacted to participate in a one-to-one telephone interview. A consent form and information letter (Appendix 15) will be sent by e-mail. If written consent is not provided, consent will be taken verbally ahead of the interview. Questions (Appendix 16) will focus on how they developed their program, and lessons learned for other remote monitoring programs. All interviews will be audio recorded and stored on a secure server at WCH, only accessible to the evaluation team. No reimbursement will be provided for their time. These interviews will take place after many of the internal (provider, patient and stakeholder) interviews and focus groups have been completed.

7. ***Collection of Process Measures:*** Every 2 weeks, process measures (Appendix 18) will be collected to understand the number of patients in the program, number of appointments etc.. This data will be securely provided to the Research Assistant to be stored in the secure folder on the WCH server.

Consent Processes

Patient Experience Survey, Interview and Chart Review Consent:

During a clinical visit with the NP or RN near the end of their time with the program, all patients will be asked (Appendix 1) if they agree to receive more information about being involved in an evaluation to help improve the program. If agreed, the Research Assistant will send an e-mail explaining the survey, interview and chart review, as well as attach a form with consent details (Appendix 2, 3). The e-mail will indicate that an RA can call them to talk through the process if further information is requested or to complete the survey over the phone. The e-mail will include a link to a RedCap survey, which will include digital consent before the survey questions are provided. If phone is preferred, the RA will complete the verbal consent and survey over the phone, and the RA will transfer the information into the RedCap survey, so all information remains in one place. If created, the written copy of responses will be destroyed as soon as it is entered into RedCap. Two reminder e-mails will be sent for those who requested e-mail follow-up, and 3 reminder phone calls for those who requested the survey by phone.

At the end of the survey (phone and e-mail), the patient will be asked if they are interested in participating in a virtual interview to further explain their overall experience with the program. They will be informed that not all participants will be contacted for an interview. Before the interview, an information letter (Appendix 5a) will be sent with the opportunity to ask questions. Once they preliminarily agree to participate, a verbal consent process (Appendix 5b) will be used to ensure consent of the interview and remind them about the recorder and their option to decline any questions. Participants will have an opportunity to ask questions before beginning. Participant will also be reminded not to provide individual names, and if they have specific concerns or comments about an individual, they will be directed to Patient Relations to follow the standardize reporting system of the hospital.

In the initial information letter, participants will be provided information about the chart review that will link their survey responses to specific information in their chart including: age, sex, comorbidities, medications, if they are registered with a PCP (Yes/No), date of first / last appointment, total number of appointments, if an oximeter was sent, if they interacted with allied health (social worked, pharmacist etc.), and number of GIM consults. The letter will explain why this information is important for our evaluation. If they do not consent to this process, they will be thanked for their time and not asked to complete the survey.

Update: All patients will have received the information letter by e-mail. Prior to starting the survey/interview, participants will have the opportunity to ask question, and verbal consent will be obtained.

Provider Surveys (All surveys): A brief survey is being sent to all physicians, nurses, and other clinicians involved in the program. Details of the study will be provided in an e-mail, and brief statement of implied digital consent will be included before the survey questions (Appendix 8). It will be made clear that this survey is voluntary, and they do not need to answer the questions. Residents are not required to complete this survey as they are part of a separate evaluation process, however they will still receive the consent information with this link and the option to complete.

Provider Interviews + Focus Groups: A Research Assistant will e-mail all providers an information letter (Appendix 7) to recruit providers for focus groups or interviews. All participants will be encouraged to read the information letter and reply to the e-mail from the Research Assistant indicating their consent. For focus groups, written consent must be submitted prior to the discussion. For interviews, written consent is encouraged, however if it is not obtained prior to the interview, verbal consent will be obtained. All consent forms/e-mail responses will be saved and kept on secure WCH drive, separately from the recordings.

Group Meeting Observation: Once per week, a member of the evaluation team will observe the huddles, and complete a standardized feedback recording sheet (Appendix 12) to take note of how decisions are made, and how the group adapts to system, process and clinical uncertainty. This observation process is described in the initial consent information for which clinicians will consent as part of involvement with the overall evaluation. At the beginning of each group meeting, the lead of the meeting will inform the group that the observer is present and can be asked to leave at any time.

Internal and External Stakeholder Interviews: A Research Assistant or Postdoctoral Researcher will e-mail all stakeholders (internal and external) an information letter and consent form (Appendix 13, 15) to recruit for interviews. All participants will be encouraged to read the information, sign the consent form and return to the WIHV Research Assistant or Postdoctoral Researcher prior to the interview, or replying to the e-mail to indicate they consent. Written consent is encouraged, however if it is not obtained prior to the interview, verbal consent will be obtained prior to the interview. All consent forms will be kept on secure WCH drive, separately from the recordings.

Analysis

As this is a developmental evaluation of a new and evolving program, all information will be used to inform the program development. Information will also be used to develop a “timeline” of the program and how it evolved since conception, using perspectives from patients, providers and stakeholders to inform the progress. Surveys will be analysed descriptively. Interviews will be analysed inductively with preliminary findings used to inform program development, and further thematic analysis undertaken to identify key themes from the data. Internal Stakeholder Interviews will be deductively analysed following the NASSS (non-adoption, abandonment, scale-up, spread, sustainability) framework.

Ethical Considerations

Participants will include patients, providers, and stakeholders. We anticipate limited risks for participants in this project. All participants will be fully advised on the nature and scope of the evaluation project as part of their participation in the COVIDCare@Home intervention. All data from participants will be collected and will be kept confidential as per the risk mitigation strategies outlined in the previous sections thereby limiting any potential privacy risks to

participants. Linking of survey and chart data is necessary to provide a comprehensive view of the program and allow for actionable change as part of this developmental evaluation. Not linking will limit the utility of the information. The only way to reliably link this information in a way that is easy for the EPIC data extracts to use, is through the MRN. Although the challenges with this linking are acknowledged, all security measures will be used (i.e. master linking logs, only kept in a password protected file, on a secure drive at WCH, only accessible to key members of the evaluation team), and all effort will be in place to ensure these are not shared beyond the required use.
