# Supplementary figures and images for: Implementation and Evaluation of COVIDCare@Home, a Family Medicine–Led Remote Monitoring Program for Patients With COVID-19: Multimethod Cross-sectional Study
Source: JMIR Hum Factors. 2022 Jun 28;9(2):e35091. doi: 10.2196/35091 (PMC9239565; doi:10.2196/35091)

## Slide 1
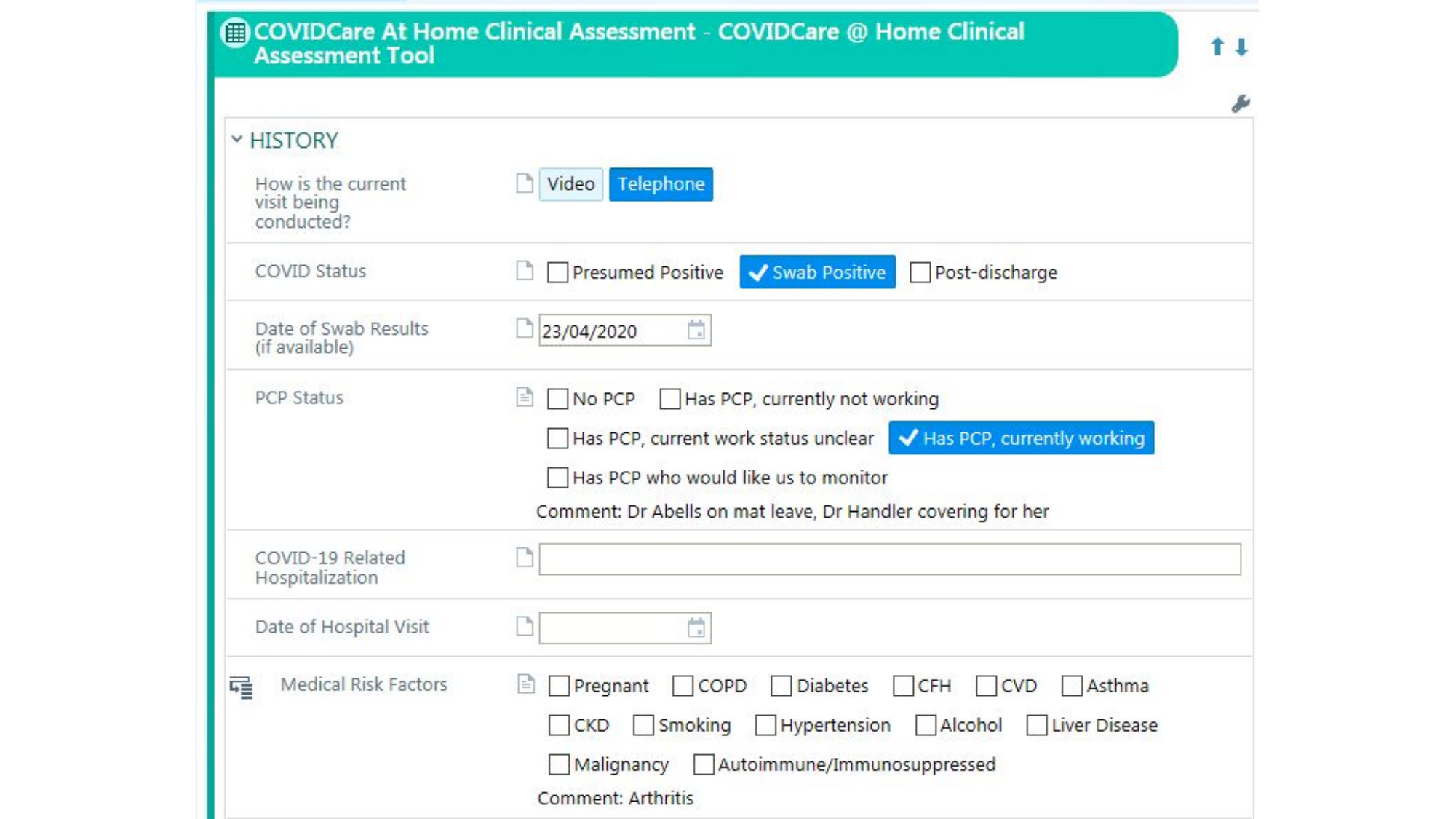

## Slide 2
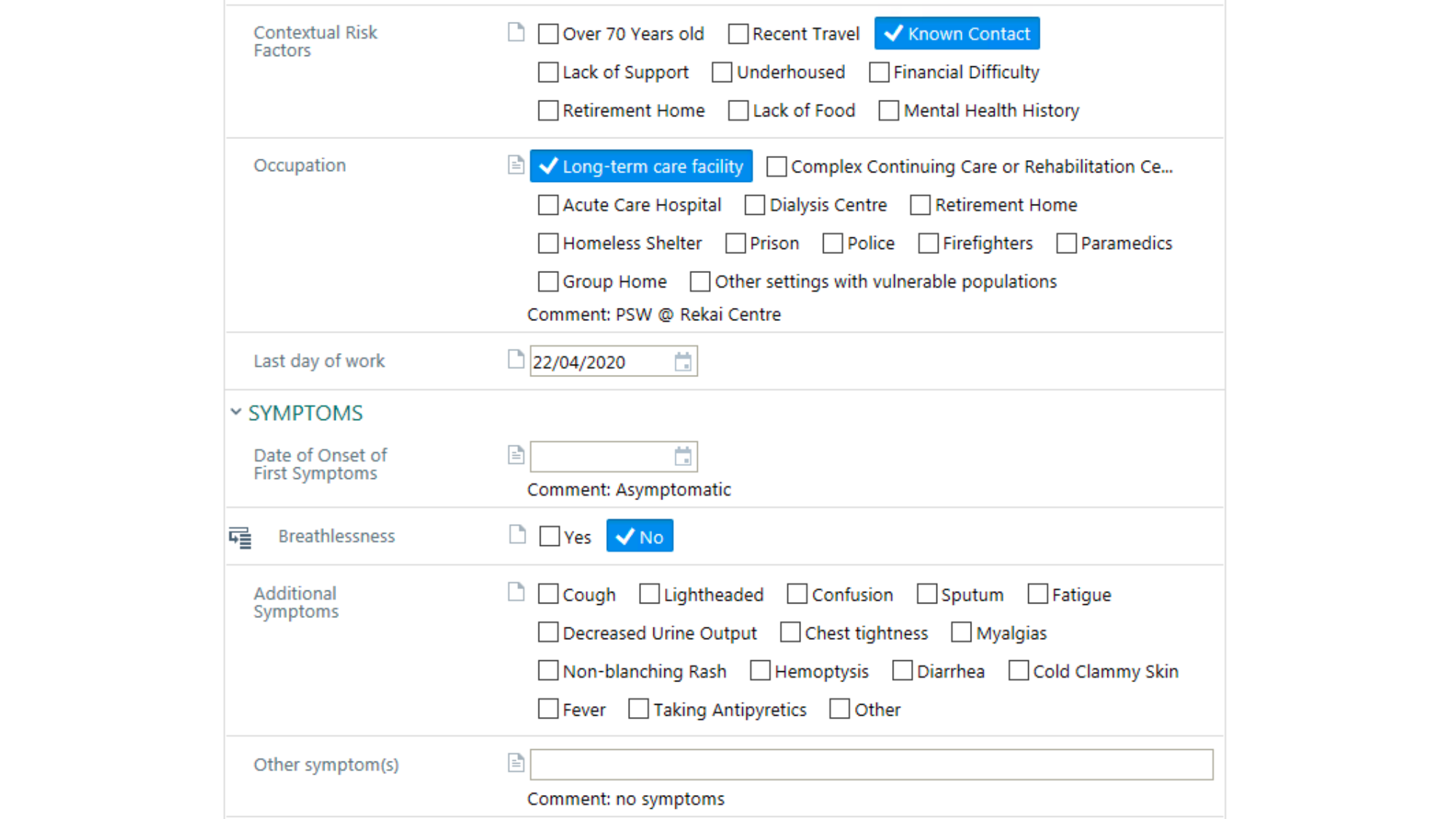

## Slide 3
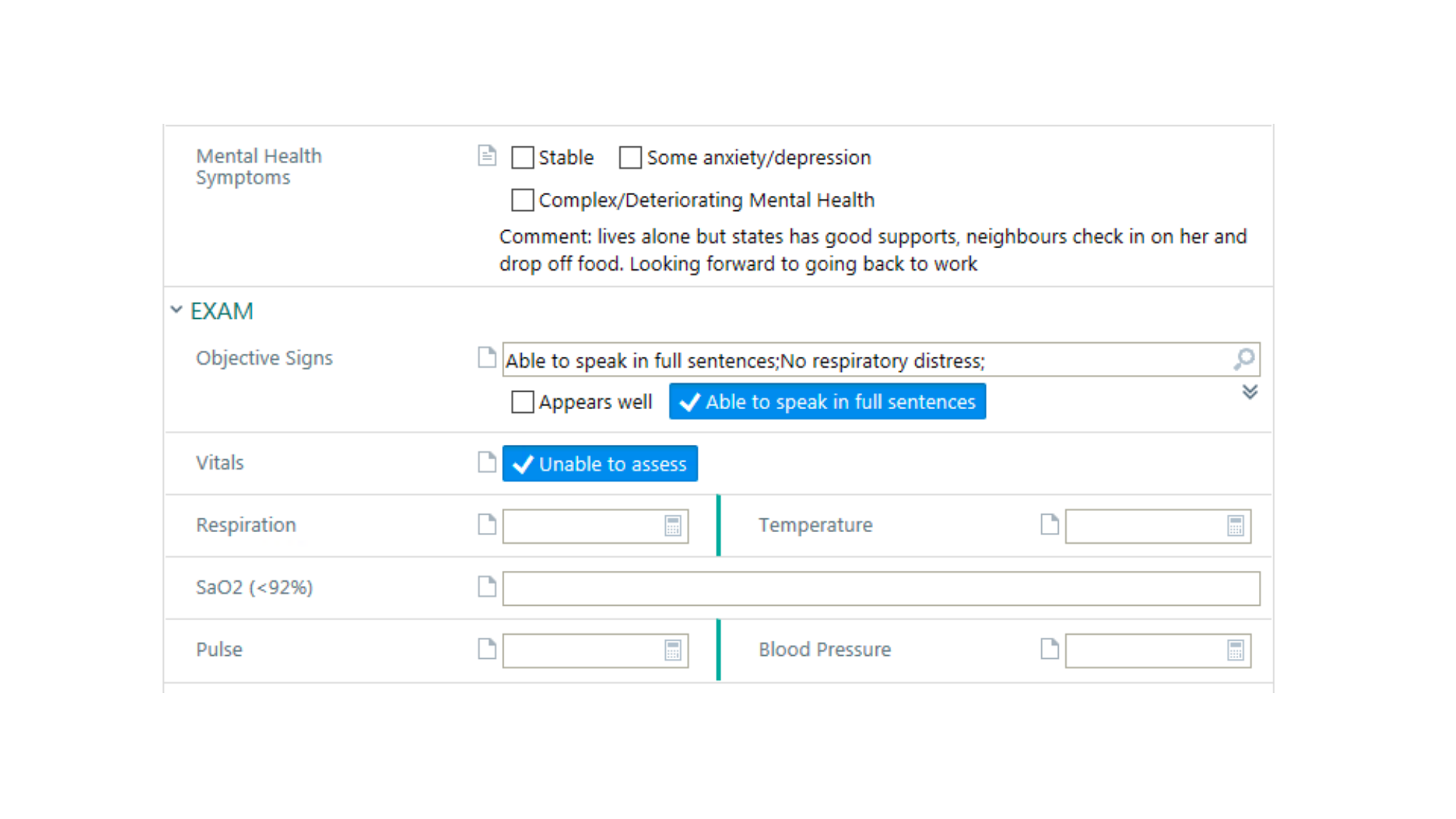

#

## Slide 4
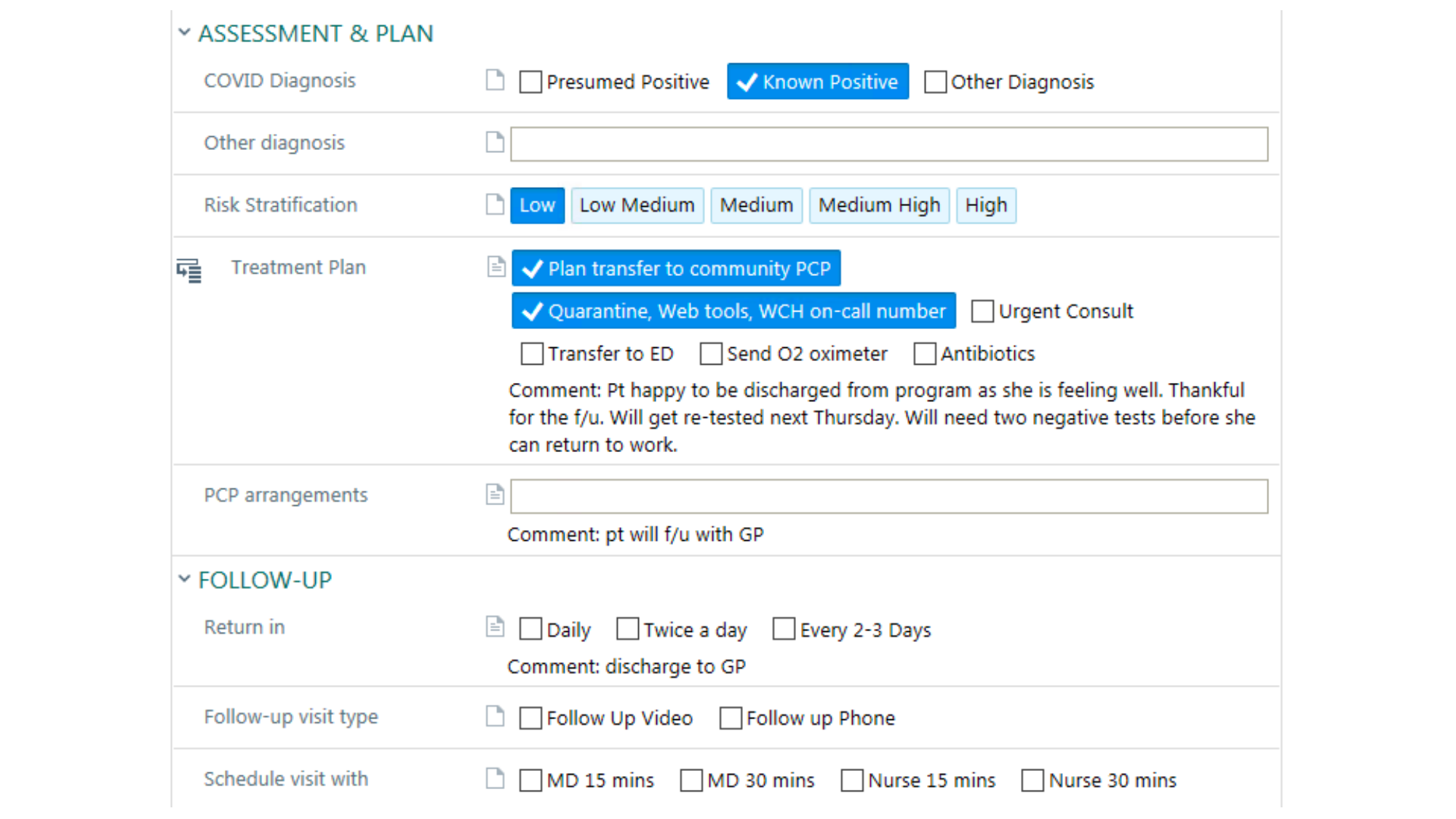

#

Supplement: Multimedia Appendix 2 [file humanfactors_v9i2e35091_app2.pptx]

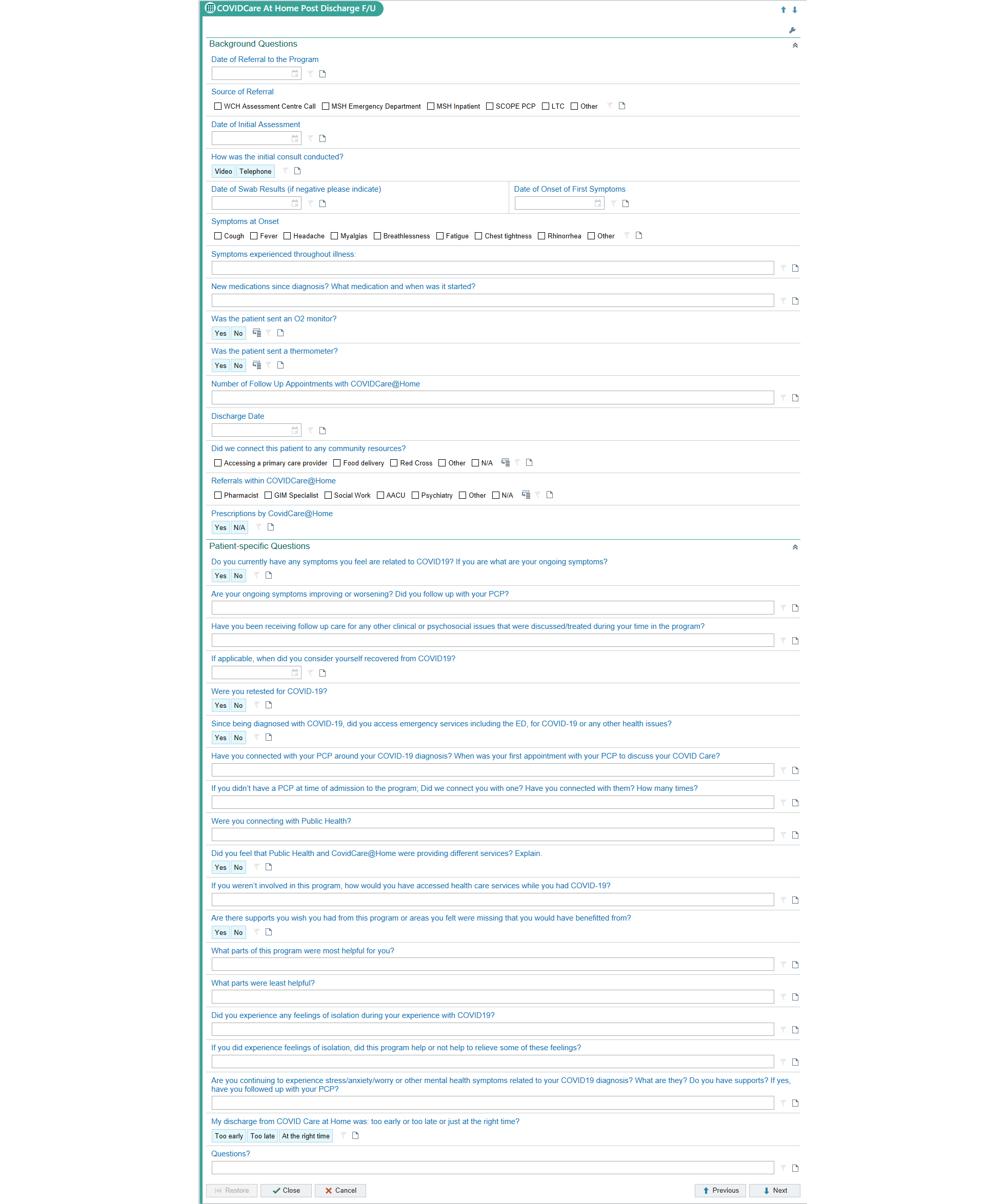

Supplement: Multimedia Appendix 3 [file humanfactors_v9i2e35091_app3.png]
