# Supplementary material for: Implementation and Evaluation of COVIDCare@Home, a Family Medicine–Led Remote Monitoring Program for Patients With COVID-19: Multimethod Cross-sectional Study
Source: JMIR Hum Factors. 2022 Jun 28;9(2):e35091. doi: 10.2196/35091 (PMC9239565; doi:10.2196/35091)
Supplement: Multimedia Appendix 4 [file humanfactors_v9i2e35091_app4.pdf]

Patient Experience Survey + Digital Consent

[The survey will be sent by e-mail using RedCap, an online survey program approved by the hospital for conducting patient surveys.]

[Digital Consent:]

WCH Logo

We are inviting you to complete this survey so we can find ways to improve how we care for patients based on your experience with the COVIDCare@Home program. You do not have to participate. You may skip any question you choose not to answer. Your answers will be kept CONFIDENTIAL. Your care at Women's College Hospital will not be affected by your decision to complete the survey.

By completing the survey, you are giving your consent for:

1) Your answers to be used for research and quality improvement purposes.

   AND

2) Your survey answers to be connected to information previously collected through the COVIDCare@Home program (information listed below and in the consent form).

You can request that we stop sending you the surveys by contacting the Research Assistant, [name].

Please complete the following statement:

I have reviewed the information provided about this study and had my questions answered. I consent to participate in this survey and to have my data (age, gender, other health conditions, medications, if you are registered with a Primary Care Provider (i.e. family doctor), date of first / last appointment, total number of appointments, if you received an oximeter, and if you met with a social worker, pharmacist, etc. or General Internal Medicine) linked to my survey response.

Yes [continue to survey questions]

No (Thank you for your time. [Close survey])

Thank you for taking the time to complete this survey.

Please answer all questions about the care you received while in the COVIDCare@Home program. If you are still involved with the program, please answer based on your experience so far.

About your experience with the COVIDCare@Home program:

Question	Response
To date, how many visits have you had with your healthcare team through telephone or video visits in this program?	1-3, 4-6, 7-9, 10-12, 13-15, 16+
This program eased my anxiety immediately after my COVID diagnosis.	Strongly Agree, Agree, Neutral, Disagree, Strongly Disagree, Not applicable (no anxiety)
I feel my needs were addressed in this program.	Strongly Agree, Agree, Neutral, Disagree, Strongly Disagree
I feel my COVID infection was well treated.	Strongly Agree, Agree, Neutral, Disagree, Strongly Disagree
I feel I had enough time with the doctor(s).	Strongly Agree, Agree, Neutral, Disagree, Strongly Disagree
I feel I had enough time with the other providers (i.e. nurse, social worker, etc.).	Strongly Agree, Agree, Neutral, Disagree, Strongly Disagree, Not applicable, Did not see other providers
I feel my care was increased when needed.	Strongly Agree, Agree, Neutral, Disagree, Strongly Disagree, Not Needed
I feel the care I received is in line with my goals and preferences.	Strongly Agree, Agree, Neutral, Disagree, Strongly Disagree
I feel I was involved in treatment decisions in a way that met my needs.	Always, Often, Sometimes, Never
I have been referred to professionals and resources (social work, pharmacist etc.) to meet my needs.	Always, Often, Sometimes, Never, Not Needed
Do you have any additional comments about your experience with the program?	[Open text]

Your Appointments

Question	Response
This program made it easy for me to see a healthcare provider immediately after my COVID diagnosis.	Strongly Agree, Agree, Neutral, Disagree, Strongly Disagree
The information provided through this program was useful for managing my care and treatment.	Strongly Agree, Agree, Neutral, Disagree, Strongly Disagree
I am concerned about the privacy of my medical information in this program.	Strongly Agree, Agree, Neutral, Disagree, Strongly Disagree
After I was referred, it was easy for me to see a social worker, psychiatrist etc.	Always, Often, Sometimes, Never, Not applicable (was not referred to other providers)
Scheduling my remote visits was easy.	Always, Often, Sometimes, Never, Not applicable (did not see other providers)
When using video, I experienced technical difficulties (e.g. unexpected disconnections, loss of sound or picture, etc.).	Always, Often, Sometimes, Never, Not applicable (did not use video)
Do you have any additional comments about your appointments?	[Open text]

Did the Program work for you?

Question	Response
I spoke with different healthcare providers during the program and felt my needs were addressed in each visit.	Always, Often, Sometimes, Never,
I spoke with different healthcare providers during the program and felt my condition was treated well in each visit.	Always, Often, Sometimes, Never,
Being involved in this program eased my anxiety.	Strongly Agree, Agree, Neutral, Disagree, Strongly Disagree, Not applicable (no anxiety)
The healthcare providers had a good understanding of my medical problem(s).	Strongly Agree, Agree, Neutral, Disagree, Strongly Disagree
The program helped me to better manage my health and medical needs for COVID-19.	Strongly Agree, Agree, Neutral, Disagree, Strongly Disagree
The program helped me decide if/when I needed in-person medical care.	Strongly Agree, Agree, Neutral, Disagree, Strongly Disagree, Not applicable
The program helped me avoid going to the Emergency Department.	Strongly Agree, Agree, Neutral, Disagree, Strongly Disagree
If you did go to the Emergency Department, were you admitted to the hospital.	Yes<br>No<br>Not applicable
Do you have any additional comments about how the program worked for you?	[Open Text]

Use of Health Services during COVID-19

Please complete the following sentence: *After your COVID positive test, if you had not been part of the COVIDCare@Home program…*

Where would you have gone for care?	❑ Walk-in clinic<br>❑ My family doctor<br>❑ Emergency Department<br>❑ I would not have gone anywhere for care<br>❑ Other: ___________________
How many in-person visits do you think you would have made to a healthcare provider?	# of visits: ______
The cost of traveling to a healthcare provider (such as parking, transit), missing work and other expenses (such as childcare) would have approximately been **(per visit):**	❑ Less than $35<br>❑ $35 to $75<br>❑ $76 to $150<br>❑ $151 to $300<br>❑ More than $300
Approximately how much time would you have spent going to an in-person healthcare visit (**per visit)**?	❑ Less than 1 hour<br>❑ 1 hour<br>❑ 2 hours<br>❑ 3 hours<br>❑ 4 or more hours
Approximately how much time would your caregiver/family member/person accompanying you have spent going to an in-person healthcare visit (**per visit**)?	❑ Less than 1 hour<br>❑ 1 hour<br>❑ 2 hours<br>❑ 3 hours<br>❑ 4 or more hours
Is there anything else that we should know about the costs and cost savings of the program to you?	[Open text]

The Future of the Program

How likely are you to recommend the program to others, on a scale of 0-10?	0 (would not recommend) – 10 (would highly recommend)
I think this program could be beneficial for other patients with a lot of health issues.	Strongly Agree, Agree, Neutral, Disagree, Strongly Disagree
Do you have any additional comments about the future of the program?	[Open text]

About You

To better understand the patients who used this program, please answer the following questions. Again, your responses will be kept confidential to maintain your privacy.

**Question**	**Responses**
How old are you?	Under 18, 18-30, 31-40, 41-50, 51-60, 61-70, 71-80, 80+; Prefer not to answer
What gender do you identify with?	Man, Woman, Transgender man, Transgender women, Identity not listed: _____, Prefer not to answer
Please enter the first 3 letters of your postal code:	[open text]
Which of the following *best describes* your racial or ethnic group?	• Asian – East (eg. Chinese, Japanese, Korean)<br>• Asian – South (eg. Indian, Pakistani, Sri Lankan)<br>• Asian – South East (eg. Malaysian, Filipino, Vietnamese)<br>• Black – African (eg. Ghanaian ,Kenyan, Somali)<br>• Black – Caribbean (eg. Barbadian, Jamaican)<br>• Black – North American (eg. Canadian, American)<br>• First Nations<br>• Indian – Caribbean (eg. Guyanese with origins in India)<br>• Indigenous not included elsewhere<br>• Inuit<br>• Latin America (eg. Argentinean, Chilean, Salvadorian)<br>• Métis<br>• Middle-Eastern (eg. Egyptian, Iranian, Lebanese)<br>• White – European (eg. English, Italian, Portuguese, Russian)<br>• White – North American (eg. Canadian, American)<br>• Mixed heritage (Please Specify): ___________________<br>• Other(s) (Please Specify): ____________________<br>• Prefer not to answer<br>• Do not know

How would you rate your ability to speak and understand English?	Very well; Well; Not well; Not at all; Unsure; Prefer not to answer; Do not know
Did someone help you with the English to answer these questions?	Yes<br>No<br>Prefer not to answer
What is your highest level of completed education?	Some primary school<br>Graduated primary school (e.g. grade 8)<br>Some high school (e.g. grade 9-12)<br>Graduated high school<br>Some post secondary education (e.g. college, university)<br>Graduated post-secondary<br>Post-graduate (MSc, PhD) study or degree<br>Other (e.g. trade training with no post-secondary component): Please specify:______<br>Prefer not to answer
Who do you currently live with?	Partner or spouse<br>Other adults<br>Nobody (live alone)<br>Parents<br>Own children<br>Other children<br>Prefer not to answer
What was your total family income before taxes last year? Check one only	$0 - $29 999<br>$30 000 - $59 999<br>$60 000 - $89 999<br>$90 000 - $119 999<br>$120 000 - $149 999<br>$150 000 or more<br>Prefer not to answer<br>Do not know
In general, would you say that your health is …	Excellent, Very good, Good, Fair, or Poor, Prefer not to answer
Do you have a primary health care provider (i.e. a family doctor)?	Yes – and currently available<br>Yes - but not currently available<br>No<br>Prefer not to answer
How many *prescribed* medications were you taking when you started with the program?	No medications<br>1 medication per day<br>2-3 medications per day

	4-6 medications per day<br>7-10 medications per day<br>10+ medications per day<br>Other (please explain): _______
Are you…?	Employed or Working<br>If Employed<br>Full Time (30+ hours per week)<br>Part Time (less than 30 hours per week)<br>Casual, on-call or short-term contract<br>Seasonal<br>Working for others<br>Self-employed<br>Other (please specify): ______<br>Unemployed or out of work<br>If Unemployed:<br>Unemployed since **before** March 1, 2020 (before COVID pandemic)<br>Unemployed **after** March 1, 2020 (due to COVID pandemic)<br>Are you seeking employment:<br>Yes<br>No<br>Prefer not to answer<br>Retired<br>Prefer not to answer<br>Other: [please specify]
Please check any of the following circumstances you experienced while you were part of the COVIDCare@Home program.	Homelessness<br>Staying in a shelter<br>Housing insecurity<br>Food insecurity<br>Financial insecurity<br>Mental health challenges<br>Lack of mental health support<br>Lack of physical support (ex. caregiver/PSW support)<br>Prefer not to answer<br>Other: ______________
Were you born in Canada?	Yes<br>No<br>Prefer not to answer
If no, how long have you lived in Canada?	Less than 1 year<br>Less than 5 years

	5 - 10 years<br>11 - 20 years<br>Over 20 years
Are you interested in participating in a one-time telephone interview where we will ask for more detailed information about your experience with this program? Please note, not everyone will be contacted for an interview.	Yes<br>No
In the future, there may be additional research studies to better understand how to treat patients with COVID-19. Would you like to be contacted about the opportunity to participate in these studies?	Yes<br>No
Are you interested in receiving a summary of the results at the end of this study?	Yes<br>No

Thank you for completing this survey.

If completed by phone, the Research Assistant will answer.

This survey was completed by phone with the Research Assistant: Yes/No

An interpreter was involved in completing this survey: Yes/No
