# Supplementary material for: Implementation and Evaluation of COVIDCare@Home, a Family Medicine–Led Remote Monitoring Program for Patients With COVID-19: Multimethod Cross-sectional Study
Source: JMIR Hum Factors. 2022 Jun 28;9(2):e35091. doi: 10.2196/35091 (PMC9239565; doi:10.2196/35091)
Supplement: Multimedia Appendix 5 [file humanfactors_v9i2e35091_app5.pdf]

Provider Survey and Digital Consent

This information will go in the survey, before survey questions.

Digital Consent:

We would like you to complete this survey so that we can better understand your experience as a clinician with the COVIDCare@Home program and find ways to improve how we care for our patients and support the providers. Your participation is completely voluntary, and you may choose to skip any question you are not comfortable answering. Your answers will be kept CONFIDENTIAL. Your name will not be used and your role at Women's College Hospital will not be affected by your decision to complete the survey. By completing the survey, you are providing consent for your answers to be used for research and quality improvement purposes. You can request that we stop sending you the surveys by contacting the Research Assistant, [name].

Please complete the following statement:

I have reviewed the information provided about this study, had my questions answered, and consent to participate in this survey.

Yes [continue to survey questions]

No (Thank you for your time. [Close survey])

Thank you for taking the time to complete this survey.

Program Logistics and Clinician Support

Questions	Response
I had prior experience with providing health care through remote monitoring programs.	Strongly Agree, Agree, Neutral, Disagree, Strongly Disagree
I feel more comfortable with remote monitoring now than when I started with the program.	Strongly Agree, Agree, Neutral, Disagree, Strongly Disagree
I feel more comfortable with the technology involved in remote monitoring now than I did when I started with the program.	Strongly Agree, Agree, Neutral, Disagree, Strongly Disagree
I feel supported to cope with the clinical uncertainty of a new illness.	Strongly Agree, Agree, Neutral, Disagree, Strongly Disagree
I feel the program has improved since it began.	Strongly Agree, Agree, Neutral, Disagree, Strongly Disagree

Patient Experience

The needs of my patients within this program are being appropriately identified.	Strongly Agree, Agree, Neutral, Disagree, Strongly Disagree
The needs of my patients are being met.	Strongly Agree, Agree, Neutral, Disagree, Strongly Disagree
I can provide patient centered care through this program.	Strongly Agree, Agree, Neutral, Disagree, Strongly Disagree
I am spending an appropriate amount of time with each patient.	Strongly Agree, Agree, Neutral, Disagree, Strongly Disagree
I can escalate patient care when needed.	Strongly Agree, Agree, Neutral, Disagree, Strongly Disagree
The care I can provide through this service aligns with the goals and preferences of my patients.	Strongly Agree, Agree, Neutral, Disagree, Strongly Disagree
I am able to address issues around social determinants of health for my patients in this program.	Strongly Agree, Agree, Neutral, Disagree, Strongly Disagree
I can easily make appropriate referrals to professionals and resources (social work, pharmacist etc.).	Strongly Agree, Agree, Neutral, Disagree, Strongly Disagree, Not Needed
The program is meeting the needs of underserved populations.	Strongly Agree, Agree, Neutral, Disagree, Strongly Disagree
I feel the program has helped avoid Emergency Department visits.	Strongly Agree, Agree, Neutral, Disagree, Strongly Disagree

Demographics

How long have you been practicing?	☐ Less than 1 year<br>☐ 1-2 years<br>☐ 3-5 years<br>☐ 6-10 years<br>☐ 11-15 years<br>☐ 15+ years<br>☐ Prefer not to answer
What gender do you identify with?	Man, Woman, Transgender man, Transgender women, Identity not listed: _____, prefer not to answer

Approximately how many patients have you seen through the COVIDCare@Home?	1-5<br>6-10<br>11-15<br>16-20
What is your role?	Attending Physician<br>Resident<br>Nurse Practitioner<br>Registered Nurse<br>Social Worker<br>Pharmacist<br>Other: ___________
Are you interested in receiving a summary of the results at the end of this study?	Yes<br>No

Thank you for completing this survey.
