# Supplementary material for: Implementation and Evaluation of COVIDCare@Home, a Family Medicine–Led Remote Monitoring Program for Patients With COVID-19: Multimethod Cross-sectional Study
Source: JMIR Hum Factors. 2022 Jun 28;9(2):e35091. doi: 10.2196/35091 (PMC9239565; doi:10.2196/35091)
Supplement: Multimedia Appendix 6 [file humanfactors_v9i2e35091_app6.pdf]

General Provider Interview Questions

Hello, I'm [name], a Research Assistant at Women's College Hospital. Thank you for agreeing to participate in this focus group about your experience with the COVIDCare@Home program. You all will have received the information we sent about why we are conducting the evaluation. Do you have any questions about that information?

In order to further improve how we care for patients with COVID-19 we are interested in understanding your experience with this program. Many of you have already completed a survey for us so thank you for providing that information. Now we would like to get a better understanding of your overall experience with the program, and how it has changed over time. Please remember you do not need to participate in this focus group, you do not need to answer the questions, and we can stop at any time. Your role at Women's College Hospital will not be affected by your decision to participate. We understand that is can be difficult to keep focus group discussions confidential, however please do not repeat the views of others outside of this meeting. We will be recording the interview to make sure we document what is discussed.

Do you have any questions before we get started? Yes/No

Is it ok if I turn the recorder on now?

Program Development

1) Can you briefly tell me about your role in the COVIDCare@Home program and when you started with the program?

2) Can you tell me about your experience being part of the COVIDCare@Home program?
    a. How were you brought on board?
    b. How are decisions made?
    c. How are decisions enacted?

3) How do you think the COVIDCare@Home program adapts to the uncertainty and changing needs of the healthcare system?

4) How do you think the COVIDCare@Home program adapts to the clinical uncertainty of treating a new illness?

5) The program was developed rapidly. What are some of the challenges and facilitators to working in a program that was developed so quickly?

6) How did the *process* of remote monitoring of COVID+ patients go with your first few patients? (Please do not provide clinical details.) Probe: When was this? How did it go? Challenges?

Patient Impact

7) Do you think this program is meeting the needs of its patients? Why/how?

8) Do you think this program is meeting the needs of underserved populations? Why/how?

9) What were some of the challenges and facilitators for meeting the needs of underserved populations?

Educational Impact

10) What is your role in relation to supervising residents who work within the program?

11) Tell us about your experience delivering virtual care before the start of the COVID-19 pandemic.
    a. Do you feel prepared as a clinician **or** as a clinical team to do this well? How so?
    b. What concerns, if any, do you have about the preparation of **your clinical team** to deliver care virtually?
    c. What concerns, if any, do you have about the preparation of **your residents** to deliver care virtually?

12) How do you think the Virtual Care environment differs from the in-person environment with regard to caring for COVID patients? Probe: Are different skills required? Do different types of challenges arise?

13) What does being competent at delivering Virtual Care mean to you?

14) What do you think the educational experiences of residents in this environment thus far?
    a. Do you think the quality of their training has been either enhanced **or** compromised in any way?
    b. Is there anything you would like to see happen differently with regard to their training? *Probe:* now or in the future

Health System Impact and Future Plans

15) Do you think this program is meeting the needs of the healthcare system? Why/how?

16) What do you see for the future of this program?
    Probe: Continues for complex patients? Phased out? What would you like to see?

17) How do you think we can make this program more sustainable?

18) What do you think are key learning points for future remote monitoring programs?

19) Is there anything else I should know about remote monitoring programs?

Is there anything else I should know about the COVIDCare@Home service?
