# Supplementary material for: Implementation and Evaluation of COVIDCare@Home, a Family Medicine–Led Remote Monitoring Program for Patients With COVID-19: Multimethod Cross-sectional Study
Source: JMIR Hum Factors. 2022 Jun 28;9(2):e35091. doi: 10.2196/35091 (PMC9239565; doi:10.2196/35091)
Supplement: Multimedia Appendix 7 [file humanfactors_v9i2e35091_app7.pdf]

Internal Stakeholder Interview Questions

Hello, I'm [name]. Thank you for agreeing to participate in this interview about your experience with the COVIDCare@Home program. You all will have received the information we sent about why we are conducting the evaluation. Do you have any questions about that information?

In order to further improve how we care for our patients we are interested in understanding your experience with the COVIDCare@Home program and how we can support other programs to be used in other locations. We would like to get a better understanding of your overall experience with the program, and how it has changed over time. Please remember you do not need to participate in this interview, you do not need to answer the questions, and we can stop at any time. Your role at Women's College Hospital will not be affected by your decision to participate. We will be recording the interview to make sure we document what is discussed.

Do you have any questions before we get started? Yes/No

Is it ok if I turn the recorder on now?

COVIDCare@Home

Program Development

1) Can you briefly tell me about your role in the COVIDCare@Home program and when you started with the program? *(Adopter System – Staff)*

2) Can you tell me about your experience being part of the COVIDCare@Home program?
    a. How did you get involved? *(Adopter System – Staff)*
    b. How are decisions made? (*(Adopter System – Staff)*)
    c. How are decisions enacted? *(Health/care Organisation)*

3) How do you think the COVIDCare@Home program adapts to the uncertainty and changing needs of the healthcare system? *(Conditions + Technology + Health/care organisation)*

4) How do you think the COVIDCare@Home program adapts to the clinical uncertainty of treating a new illness? (Condition + Health/care Organisation)

5) The program was developed rapidly. What are some of the challenges and facilitators to working in a program that was developed so quickly? *(Wider system + Continuous embedding and adoption)*

Patient Impact

6) Do you think this program is meeting the needs of its patients? Why/how? *(Adopter system – patient)*

7) Do you think this program is meeting the needs of underserved populations? Why/how? *(Adopter system – patient + Value Proposition)*

8) What were some of the challenges and facilitators for meeting the needs of underserved populations? *(Adopter system – patient + Value Proposition + Wider System)*

Health System Impact and Future Plans

9) Do you think this program is meeting the needs of the healthcare system? Why/how? *(Wider system)*

10) What do you see for the future of this program? *(Wider system + Continuous embedding and adoption)* Probe: Continues for complex patients? Phased out? What would you like to see?

11) Is there anything else I should know about the COVIDCare@Home service?
