# Supplementary material for: Implementation and Evaluation of COVIDCare@Home, a Family Medicine–Led Remote Monitoring Program for Patients With COVID-19: Multimethod Cross-sectional Study
Source: JMIR Hum Factors. 2022 Jun 28;9(2):e35091. doi: 10.2196/35091 (PMC9239565; doi:10.2196/35091)
Supplement: Multimedia Appendix 8 [file humanfactors_v9i2e35091_app8.pdf]

Supplemental Table 1: *Clinical and Service Utilization Data:* Demographic and clinical characteristics of all patients admitted to the program between April 8-December 8, 2021, including Adoption and Feasibility Outcomes.

	**Number (%) of total patients**
**Demographics**	
Number of patients	616
Age	
Median age and IQR	35 (IQR=25)
Under 18 years of age	23 (4%)
Over 60 years of age	85 (14%)
Sex	
Man	279 (45%)
Woman	337 (55%)
**Clinical Characteristics**	
Co-morbidities	
Asthma	41 (7%)
Autoimmune/Immunosuppressed	13 (2%)
Congestive Heart Failure	1 (0%)
Liver disease	3 (0%)
Chronic Obstructive Pulmonary Disease	3 (0%)
Cardiovascular disease	3 (0%)
Diabetes	36 (6%)
Hypertension	34 (6%)
Malignancy	2 (0%)
Anxiety/Depression	33 (5%)
Dyslipidemia	20 (3%)
Other Factors	
Smoking~	47 (8%)
Pregnancy+	5 (1%)
**COVID-19 Characteristics**	
COVID-19 Status	
Known Positive	539 (88%)
Presumed Positive	14 (2%)
Other Diagnosis	5 (1%)
Null/Missing	58 (9%)
Identified Risk Factors for Acquiring COVID	
Occupation:	
Long-term care home	13 (2%)
Acute Care	4 (1%)
Shelter	7 (1%)
Complex Continuing Care	9 (1%)
Grocery Store	1 (0%)
Other setting with vulnerable populations	6 (1%)
Null/Missing	576 (94%)

	Number (%) of total patients
Recent Travel	25 (4%)
Financial Difficulty	8 (1%)
Known Contact	213 (35%)
Lack of Support	6 (1%)
Underhoused	9 (1%)
Lack of Food	3 (1%)
Mental Health History	2 (0%)
Retirement Home	1 (0%)
**Access to Care**	
Has a Primary Care Provider (PCP)*	357 (58%)
Would like to be monitored by PCP	5
Current work status of PCP unclear	67
PCP not currently working	10
PCP currently working	278
No PCP	171 (28%)
Null/Missing	88 (14%)
**Process Outcomes**	
Adoption & Feasibility	
Number of visits	
Total	3412 visits
Generic Provider	689 visits
Family Physician Staff/Resident	2114 visits
Registered Nurse	439 visits
Advanced Nurse	2 visits
Social Work/Mental Health	149 visits
Pharmacist	19 visits
Median number of visits per patient	5 visits (IQR=4)
Median time from swab results to first visit	3 days (IQR=3)
Median length of follow-up in program^	7 days (IQR=27)

PCP: Primary Care Providers

* Can select more than one option

~ Any current use of tobacco cigarettes

+ Self-identified by the patient

^ Length of follow-up is the time from the first appointment to the last.

Supplemental Table 2: *Patient Post-Discharge Surveys:* Demographics, clinical risk factors, implementation and quality outcomes collected from patients approximately 2-weeks post-discharge.

Questions	Number (%) of Patients
Number of patients	194
Sex	
Female	110 (57%)
Male	84 (43%)
Age	
Median age and IQR	35 (IQR=25)
Under 18 years of age	1 (1%)
Over 60 years of age	23 (12%)
Co-morbidities	
Asthma	17 (9%)
Autoimmune/Immunosuppressed	5 (3%)
Liver disease	3 (2%)
Cardiovascular disease	1 (1%)
Diabetes	8 (4%)
Hypertension	11 (6%)
Anxiety/Depression	8 (4%)
Dyslipidemia	2 (1%)
Other Factors	
Smoking~	9 (5%)
Pregnancy^	2 (1%)
Identified Risk Factors for Acquiring COVID	
Occupation:	
Long-term care home	9 (5%)
Acute Care	2 (1%)
Shelter	4 (2%)
Complex Continuing Care	5 (3%)
Grocery Store	1 (1%)
Other setting with vulnerable populations	2 (1%)
Null	171 (88%)
Recent Travel	14 (7%)
Financial Difficulty	3 (2%)
Known Contact	48 (25%)
Lack of Support	3 (2%)
Underhoused	4 (2%)
Lack of Food	1 (1%)
Has a Primary Care Provider*	111 (57%)
Would like to be monitored	4 (2%)
Current work status unclear	30 (15%)
Currently not working	5 (3%)

Currently working	75 (39%)
Does not have a Primary Care Provider	49 (25%)
Null/Missing	34 (18%)
Feasibility and Adoption	
Source of Referral	
WCH Assessment Centre	177 (91%)
MSH Emergency Department	3 (2%)
Other	11 (6%)
Null/Missing	3 (2%)
Sent an:	
Oximeter	39 (20%)
Thermometer	14 (7%)
Referrals within CC@H*:	
Pharmacist	8 (4%)
General Internal Medicine (GIM) Specialist	1 (0)
Social Work	60 (31%)
Acute Ambulatory Care Unit (AACU)	1 (0)
Other	2 (1%)
N/A	119 (61%)
Null/Missing	9 (5%)
Prescription by CC@H	
Yes	21 (11%)
N/A	166 (86%)
Null/Missing	7 (4%)
Effectiveness	
What parts of this program were most helpful for you?	
Regular check-ins	69
Positive care experience (i.e., helpful, warm interaction, kind providers, listening, "everything")	48
Felt supported and reassured	24
Received comprehensive service (i.e., food, equipment, and medication delivery, connected to a PCP, connected to social work, received an O2 saturation monitor).	22
Safe, timely, and personalized care (i.e., easy and timely access to care, not discharged until a PCP was available, consistent provider, hands-on care)	22
Provided advice and allowed patients to asked questions	19
Eased stress / anxiety	7
Missing (null)	108
What parts were least helpful?	
None/Nothing to change	47
Too many check-ins	3
Technology (missed calls, resident portal, wanted online chats)	3
Communication challenges (i.e., understanding provider wearing a mask;)	2

Poor coordination (with AACU, medication taking a long time)	2
Program not helpful	2
Conflicting information with Public Health	1
Resident attitudes	1
Other (financial aspect; wanted more information about medications; did not feel like talking)	3
Missing (null)	137
Are there supports you wish you had from this program or areas you felt were missing that you would have benefitted from?	
Yes	12 (6%)
No	108 (56%)
Missing (Null)	74 (38%)
Patient Centeredness	
Did you experience any feelings of isolation during your experience with COVID-19?	
Yes	23 (12%)
A little bit	9 (5%)
No	57 (29%)
Unsure	1 (0)
N/A	4 (2%)
If you did experience feelings of isolation, did this program help to relieve some of these feelings? [$]	
Yes	21 (11%)
No	7 (4%)
Unsure	3 (2%)
N/A	6 (3%)
Are you continuing to experience stress/anxiety/worry or other mental health symptoms related to your COVID-19 diagnosis?	
Yes	16 (8%)
No	52 (27%)
Unclear	5 (3%)
Population Health	
Connected to community resources:*	
Accessing a PCP	32 (17%)
Food delivery	9 (5%)
Red Cross	4 (2%)
Other	4 (2%)
N/A	139 (72%)
Null/Missing	16 (8%)
If you didn't have a PCP at time of admission to the program, did we connect you with one?	
Yes	16 (8%)
No	7 (4%)
If you weren't involved in this program, how would you have accessed health care services while you had COVID-19?	

Primary Care Provider	33 (17%)
Would not seek care	17 (9%)
Emergency Department	14 (7%)
Public Health	14 (7%)
Walk-in Clinic	4 (2%)
Telehealth	2 (1%)
Unsure	11 (6%)
Other	4 (2%)
Were you connecting with Public Health?	
Yes	50 (26%)
No	21 (11%)
Null /Missing	123 (66%)
Cost	
Emergency Service/Emergency Department Visits	10 (5%)

PCP – Primary Care Provider
* Can select more than one option
~ Any current use of tobacco cigarettes
+ Self-identified by the patient
$ Unclear if only those who experienced some feelings of isolation were asked this question.

Supplemental Table 3: *Online Patient Survey Data:* Demographics information and clinical characteristics collected through the online patient survey.

	**Number (%) for patient survey data**
Number of patients	14
Age	
Median age and IQR#	33 (IQR=21)
18-30	7 (50%)
31-40	1 (7%)
51-60	4 (29%)
61-70	1 (7%)
Missing	1 (7%)
Sex	
Man	4 (29%)
Woman	9 (64%)
Missing	1 (7%)
Location	
Toronto	12 (86%)
Vaughan	1 (7%)
Missing	1 (7%)
Racial or ethnic group	
Asian – East (eg. Chinese, Japanese, Korean)	1 (7%)

	Number (%) for patient survey data
Asian – South (eg. Indian, Pakistani, Sri Lankan)	1 (7%)
Asian – South East (eg. Malaysian, Filipino, Vietnamese)	1 (7%)
White – European (eg. English, Italian, Portuguese, Russian)	5 (36%)
White – North American (eg. Canadian, American)	4 (29%)
Mixed heritage	1 (7%)
Missing	1 (7%)
Ability to speak and understand English	
Very well	12 (86%)
Well	1 (7%)
Missing	1 (7%)
Received help with the English to answer questions	
No	13 (93%)
Prefer not to answer	1 (7%)
Highest level of education	
Some post-secondary education	3 (21%)
Graduated post-secondary	7 (50%)
Post-graduate study or degree	3 (21%)
Missing	1 (7%)
Live with:	
Partner or spouse	6 (43%)
Other adults	2 (14%)
Nobody (live alone)	4 (26%)
Parents	1 (7%)
Missing	1 (7%)
Total family income before taxes last year	
$0 - $29 999	2 (14%)
$30 000 - $59 999	1 (7%)
$60 000 - $89 999	1 (7%)
$90 000 - $119 999	3 (21%)
$120 000 - $149 999	2 (14%)
$150 000 or more	3 (21%)
Prefer not to answer	1 (7%)
Missing	1 (7%)
Employed or Working (total)	10 (71%)
Full Time (30+ hours per week)	9/10 (90%)
Part Time (less than 30 hours per week)	1/10 (10%)
Unemployed or out of work (total)	1 (7%)
Unemployed **after** March 1, 2020 (due to COVID pandemic)	1/1 (100%)
Seeking employment:	
Yes	1/1 (100%)
Prefer not to answer	1 (7%)
Missing	1 (7%)
Born in Canada	

	Number (%) for patient survey data
Yes	8 (57%)
No	5 (36%)
Missing	1 (7%)
If not born in Canada, years lived in Canada	
11 - 20 years	1/5 (20%)
Over 20 years	4/5 (80%)
Circumstances experienced while part of the program:	
Financial insecurity	2 (14%)
Mental health challenges	4 (28%)
Lack of mental health support	1 (7%)
Prefer not to answer	1 (7%)
None of the above	6 (43%)
Missing	1 (7%)
**Clinical Characteristics**	
General Health	
Excellent	3 (21%)
Very good	6 (43%)
Good	4 (28%)
Missing	1 (7%)
Medication	
No medications	9 (64%)
1 medication taken per day	1 (7%)
2-3 medications taken per day	3 (21%)
Missing	1 (7%)
Co-morbidities#	
Asthma	2 (14%)
Autoimmune/Immunosuppressed	1 (7%)
Anxiety/Depression	1 (7%)
Other Factors#	
Smoking*	1 (7%)
**Identified Risk Factors for Acquiring COVID**	
Occupation:	
Long-term care home	1 (7%)
Complex Continuing Care	1 (7%)
Null	12 (86%)
Known Contact	3 (21%)
**Access to Care**	
Has a Primary Care Provider	
Yes (currently available)	11 (79%)
Yes (not currently available)	2 (14%)
Missing	1 (7%)
To date, how many visits have you had with your healthcare team through telephone or video visits in this program?	

	Number (%) for patient survey data
1-3	1 (7%)
4-6	6 (43%)
7-9	2 (14%)
10-12	2 (14%)
13-15	1 (7%)
16+	1 (7%)

[#] Utilization data from EPIC
* Any current use of tobacco cigarettes

Supplemental Table 4: *Online Patient Survey Results:* Detailed information collected through the online patient survey focused on feasibility, adoption, safety, effectiveness, patient centeredness, and health system connection & impact.

Feasibility & Adoption						
		**Always**	**Often**	**Sometimes**	**Never**	**N/A**
Scheduling my remote visits was easy.		11 (79)	1 (7)	0	2 (14)	0
When using video, I experienced technical difficulties (e.g. unexpected disconnections, loss of sound or picture, etc.).		0	1 (7)	2 (14)	7 (50)	4 (29)
After I was referred, it was easy for me to see a social worker, psychiatrist etc.		1 (7)	1 (7)	0	0	12 (86)
	**Strongly Agree**	**Agree**	**Neutral**	**Disagree**	**Strongly Disagree**	**N/A**
This program made it easy for me to see a healthcare provider immediately after my COVID diagnosis.	6 (43)	5 (36)	3 (21)	0	0	0
Safety						
		**Always**	**Often**	**Sometimes**	**Never**	**N/A**
I spoke with different healthcare providers during the program and felt my condition was treated well in each visit.		7 (50)	4 (29)	1 (7)	0	2 (14)

	**Strongly Agree**	**Agree**	**Neutral**	**Disagree**	**Strongly Disagree**	**N/A**
I feel my COVID infection was well treated.	7 (50)	3 (21)	4 (29)	0	0	0
The healthcare providers had a good understanding of my medical problem(s).	7 (50)	4 (29)	2 (14)	1 (7)	0	0
I feel my care was increased when needed.	5 (36)	4 (29)	1 (7)	1 (0)	0	3 (21)
The program helped me decide if/when I needed in-person medical care.	5 (36)	4 (29)	0	0	1 (7)	4 (29)
The program helped me avoid going to the Emergency Department. *(Note: no patient who answered the survey went to hospital)*	8 (57)	3 (21)	3 (21)	0	0	0
Effectiveness						
		**Always**	**Often**	**Sometimes**	**Never**	**N/A**
I was referred to professionals and resources (social work, pharmacist etc.) to meet my needs.		3 (21)	1 (7)	1 (7)	0	9 (64)
	**Strongly Agree**	**Agree**	**Neutral**	**Disagree**	**Strongly Disagree**	**N/A**
The program helped me to better manage my health and medical needs for COVID-19.	8 (57)	3 (21)	2 (14)	1 (7)	0	0

The information provided through this program was useful for managing my care and treatment.	7 (50)	4 (29)	2 (14)	1 (7)	0	0
I feel I had enough time with the doctor(s).	7 (50)	5 (36)	2 (14)	0	0	0
I feel I had enough time with the other providers (i.e. nurse, social worker, etc.).	4 (29)	5 (36)	4 (29)	1 (7)	0	0
Patient Centeredness		**Always**	**Often**	**Sometimes**	**Never**	**N/A**
I spoke with different healthcare providers during the program and felt my needs were addressed in each visit.		8 (57)	3 (21)	2 (14)	1 (7)	0
I feel I was involved in treatment decisions in a way that met my needs.		9 (64)	2 (14)	2 (14)	1 (7)	0
	**Strongly Agree**	**Agree**	**Neutral**	**Disagree**	**Strongly Disagree**	**N/A**
I feel the care I received is in line with my goals and preferences.	7 (50)	6 (43)	0	1 (7)	0	0
I feel my needs were addressed in this program.	8 (57)	5 (36)	1 (7)	0	0	0
This program eased my anxiety immediately after my positive COVID test.	7 (50)	4 (29)	2 (14)	1 (7)	0	0

Being involved in this program eased my anxiety.	7 (50)	4 (29)	1 (7)	0	0	2 (14)
Population Health						
	**Strongly Agree**	**Agree**	**Neutral**	**Disagree**	**Strongly Disagree**	**N/A**
I think this program could be beneficial for other patients with a lot of health issues.*	9 (64)	1 (7)	1 (7)	1 (7)	0	0
I am concerned about the privacy of my medical information in this program.	0	2 (14)	3 (21)	3 (21)	6 (42)	0

**2 (14%) missing data*
*** 1 (7%) missing data*

Where would you have gone for care?**	
Walk-in clinic	1 (7%)
My family doctor	8 (57%)
Emergency Department	4 (29%)
I would not have gone anywhere for care	2 (14%)

How many in-person visits do you think you would have made to a healthcare provider?	Range: 0 – 25<br>Mean: 3.15<br>SD: 6.74

Cost

Cost if would have had to travel to a healthcare provider (i.e., parking, transit), missing work and other expenses (i.e., childcare) (per visit):**	
Less than $35	9 (64%)
$35 to $75	0
$76 to $150	0
$151 to $300	1 (7%)

More than $300	3 (21%)
Time spent if would have needed to go to an in-person healthcare visit (per visit)**	
Less than 1 hour	1 (7%)
1 hour	3 (21%)
2 hours	6 (43%)
3 hours	2 (14%)
4 or more hours	1 (7%)
Time spent by caregiver/family member/person accompanying patient if needed to attend an in-person healthcare visit (per visit)**	
Less than 1 hour	7 (50%)
1 hour	2 (14%)
2 hours	2 (14%)
3 hours	2 (14%)
4 or more hours	0
