# Supplementary material for: Implementation and Evaluation of COVIDCare@Home, a Family Medicine–Led Remote Monitoring Program for Patients With COVID-19: Multimethod Cross-sectional Study
Source: JMIR Hum Factors. 2022 Jun 28;9(2):e35091. doi: 10.2196/35091 (PMC9239565; doi:10.2196/35091)
Supplement: Multimedia Appendix 9 [file humanfactors_v9i2e35091_app9.pdf]

Provider Demographics and Results

Supplementary Table 5: *Provider Survey Demographics:* Demographic from provider survey responses combined for all 3 rounds. Round 1: June 24-July 21, 2020; Round 2: August 24-26, 2020; Round 3: December 2-9, 2020.

Demographics	Number of providers
Total # of Providers who completed the survey	22
**Gender**	
Female	21 (95%)
Non-Binary	1 (5%)
**Current Role**	
Attending Physician	8 (36%)
Nurse Practitioner	2 (9%)
Registered Nurse	3 (14%
Social Worker	5 (23%)
Pharmacist	2 (9%)
Other (unspecified)	2 (9%)
**Years in Practice**	
Less than 1 year	1 (5%)
1-2 years	3 (14%)
6-10 years	7 (32%)
11-15 years	4 (18%)
16+ years	6 (27%)
**Patients Seen in the Program**	
16-20 patients	2 (9%)
21-30 patients	3 (14%)
31-40 patients	4 (14%)
41-50 patients	1 (5%)
51+ patients	12 (55%)

Supplementary Table 6: *Provider Survey Results:* Provider survey response focused on feasibility, adoption, safety, equity, effectiveness, patient centeredness, and cost. Round 1: June 24-July 21, 2020; Round 2: August 24-26, 2020; Round 3: December 2-9, 2020.

**Questions**	**Round R1=10 R2 = 6 R3 = 6**	**Agree/Strongly Agree (n)**	**Neutral (n)**	**Disagree/Strongly Disagree (n)**
Feasibility and Adoption				
I had prior experience with providing health care through remote monitoring programs.	R1<br>R2<br>R3	1<br>4<br>1	1<br>0<br>0	8<br>2<br>5
I feel more comfortable with remote monitoring now than when I started with the program.	R1<br>R2<br>R3	10<br>4<br>6	0<br>2<br>0	0<br>0<br>0
I feel more comfortable with the technology involved in remote monitoring now than I did when I started with the program.	R1<br>R2<br>R3	9<br>4<br>4	0<br>2<br>1	1<br>0<br>1
I can easily make appropriate referrals to professionals and resources (social work, pharmacist etc.).	R1<br>R2*<br>R3	9<br>5<br>6	0<br>0<br>0	1<br>0<br>0
Safety				
I feel supported to manage the clinical uncertainty of a new illness.	R1<br>R2<br>R3	10<br>5<br>2	0<br>1<br>2	0<br>0<br>1
I can escalate patient care when needed.	R1<br>R2<br>R3	9<br>6<br>5	0<br>0<br>1	1<br>0<br>0
Equity				
I am able to address issues around social determinants of health for my patients in this program.	R1<br>R2<br>R3	9<br>5<br>3	0<br>1<br>3	1<br>0<br>0
The program is meeting the needs of underserved populations.	R1<br>R2*<br>R3	9<br>4<br>1	1<br>1<br>2	0<br>0<br>3
Effectiveness				
I feel the program has improved since it began.	R1<br>R2*<br>R3	10<br>4<br>3	0<br>1<br>2	0<br>0<br>1
I am spending an appropriate amount of time with each patient.	R1<br>R2	10<br>6	0<br>0	0<br>0

	R3	4	1	1
Patient Centeredness				
The needs of my patients within this program are being appropriately *identified*.	R1<br>R2<br>R3	9<br>6<br>4	1<br>0<br>1	0<br>0<br>1
The needs of my patients are being *met*.	R1<br>R2<br>R3	9<br>5<br>5	1<br>1<br>1	0<br>0<br>0
I can provide patient centered care through this program.	R1<br>R2<br>R3	8<br>6<br>5	2<br>0<br>1	0<br>0<br>0
The care I can provide through this service aligns with the goals and preferences of my patients.	R1<br>R2<br>R3	6<br>6<br>5	2<br>0<br>1	2<br>0<br>0
Cost				
I feel the program has helped avoid Emergency Department visits.	R1<br>R2<br>R3	9<br>5<br>4	1<br>1<br>2	0<br>0<br>0

* 1 missing
